# Biogas Could Enable Cost-Effective Defossilization of *n*‑Propanol and Its Derivatives

**DOI:** 10.1021/acssuschemeng.5c13160

**Published:** 2026-03-20

**Authors:** Abhinandan Nabera, Sachin Jog, Juan D. Medrano-García, Robert Istrate, Gonzalo Guillén-Gosálbez

**Affiliations:** a Department of Chemistry and Applied Biosciences, Institute for Chemical and Bioengineering, 27219 ETH Zurich, Vladimir Prelog Weg 1, Zurich 8093, Switzerland; b NCCR Catalysis, Zurich 8093, Switzerland; c Institute of Environmental Sciences (CML), 4496 Leiden University, Einsteinweg 2, 2333 CC Leiden, The Netherlands

**Keywords:** carbon capture and utilization, reverse water-gas shift, renewable carbon, biogas reforming, catalytic hydrogenation

## Abstract

Defossilization routes for chemicals often entail a large economic premium, hampering their potential adoption. Using techno-economic analysis and life cycle assessment, we show that biogas-based routes for *n*-propanol synthesis could be economically, in addition to environmentally, appealing. Specifically, we find that the biogas-based propanol route (using biogas, electrolytic hydrogen, and fossil ethylene) could become a win–win alternative, with 30% better economic performance and 70–73% lower climate change impacts than the fossil analog while not entailing any significant burden-shifting. Overall, our results highlight the promising use of biogas as an alternative feedstock in the conventional hydroformylation–hydrogenation process for cost-effective, low-carbon *n*-propanol production.

## Graphical abstract

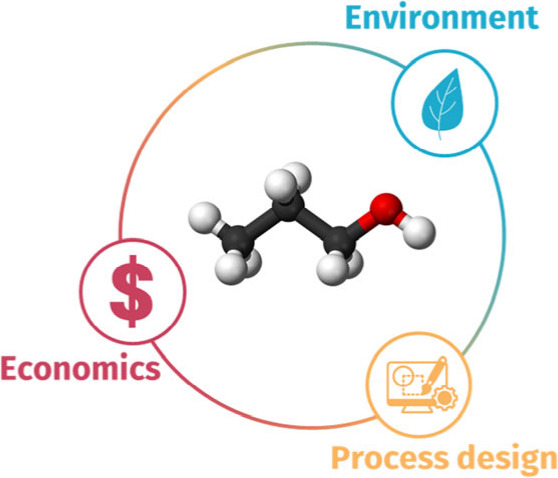

## Introduction

The heavy reliance on fossil fuels has led the chemical industry to contribute approximately 10% of global anthropogenic CO_2_ emissions.^1^ Furthermore, the demand for chemicals is projected to rise significantly in the future (e.g., two- to five-fold from 2020 to 2050).^2,3^ To meet recent climate pacts such as the Paris Agreement^4^ and the Glasgow Climate Pact,^5^ and to enhance strategic autonomy via carbon circularity, the chemical industry needs to adopt more sustainable practices. Thus, shifting from fossil fuels toward low-carbon feedstocks through carbon capture and utilization (CCU), and biomass and waste use has been gaining attention, especially in the production of platform chemicals.^6–8^

In this context, n-propanol (referred to as propanol in the rest of the article) is an important platform chemical, widely used as an additive in the cosmetics and pharmaceutical industries,^9^ and also as a precursor for the production of amines, esters, and ethers.^10,11^ With an annual demand of 4 Mt and a growth rate of 5%,^12^ propanol is primarily produced following a multistep process involving hydroformylation of syngas (derived from natural gas reforming) and ethylene (derived from naphtha cracking). The resulting propanal undergoes hydrogenation using hydrogen obtained from natural gas reforming to yield propanol. This reliance on fossil fuel-based precursors results in a high carbon footprint of around 3.1 kg CO_2_e kg^–1^ propanol.^11^ One alternative approach is to resort to one-step synthesis routes based on thermocatalysis,^12,13^ electrocatalysis,^14^ and fermentation,^10^ which can convert CO_2_ (or syngas) into propanol in a single reaction step. However, the low technology readiness level (TRL) of these technologies makes the decarbonization of the conventional fossil multistep process as the currently preferred choice,^15^ thus making the defossilization of the two main precursors, i.e., ethylene and syngas, essential to reduce the footprint of such two-step route.

With regards to the ethylene feedstock used in propanal production (prior to its subsequent transformation to propanol), the use of low-carbon methanol-to-olefins (MTO) has emerged as a promising production route. More specifically, the MTO route could use captured CO_2_ and electrolytic hydrogen to produce methanol, which could then be employed to produce olefins such as ethylene, propylene, and butene. For syngas, there are various options for defossilization. One such route utilizes electrolytic hydrogen (produced via renewable energy-powered electrolysis) and CO_2_ from direct air capture (DAC) in a reverse water-gas shift (RWGS) reaction to produce syngas.^16^ Alternatively, syngas could be produced through the reforming of other methane sources such as biogas, a renewable gaseous fuel^17^ obtained by biodegradation of organic materials such as food waste, sewage sludge, and agricultural residues.^18^ As such, these low-carbon precursors can be used in alternative syngas production routes to enable low-carbon propanol production routes.

Although potentially highly promising, these routes would still require systematic life cycle assessment (LCA) and techno-economic analysis (TEA) to evaluate their cost-effectiveness in mitigating greenhouse gas (GHG) emissions and their overall environmental performance compared to the fossil-based process. However, the scope of existing literature is limited, overlooking many potential alternative decarbonization routes for propanol production. For example, Vo et al.^12^ compared the fossil-based process with an alternative process using captured CO_2_, renewable hydrogen, and fossil ethylene through TEA and LCA. They concluded that the alternative process could be profitable while reducing life cycle CO_2_ emissions by 36% (considering the full life cycle of propanol). However, the ethylene price considered in their work is about six to 11 times lower than typical costs reported in the literature (0.11 USD kg^–1^ versus 0.59–1.16 USD kg^–1^).^19,20^ Lee et al.^21^ studied various process configurations for the direct hydrogenation of CO_2_ to propanol. They observed that while this alternative process is between two and five times more expensive, the climate change impact could be reduced by 53% with gray hydrogen and 77% by using green hydrogen. Overall, these studies show that CO_2_-based routes for propanol production are either economically infeasible and/or achieve only moderate GHG emissions mitigation.

While biogas-based routes hold potential to overcome these limitations, comprehensive LCA and TEA for such routes are still largely lacking. Motte et al.^11^ considered upgrading biogas to biomethane via chemical absorption, followed by its use in propanol production, and reported cradle-to-gate climate change impacts of ∼6.0 kg CO_2_e kg^–1^ propanol (versus 3.1 kg CO_2_e kg^–1^ for the conventional fossil case reported in the same study). This suggests that upgrading-based routes can be impacted by additional energy requirements and potential methane leakage associated with the upgrading step,^8^ and motivates considering alternatives that directly convert biogas to syngas (e.g., via dry reforming), thereby improving carbon utilization by converting both CO_2_ and methane in biogas into syngas. To the best of our knowledge, no prior study has evaluated the environmental and economic potential of direct biogas-to-syngas (dry reforming) as the syngas supply route in the conventional hydroformylation-based propanol production process.

Moreover, existing studies primarily assess the environmental performance of innovative production routes under current global supply chain conditions. In practice, however, these new routes will require time to become established in the market,^22^ during which many economic sectors are expected to undergo significant transformation. For example, the GHG emissions mitigation potential and environmental impacts of a given chemical production route will likely differ in 2050 compared to today, owing to global decarbonization in the power generation sector and other industries that interact with the chemical industry and contribute to its life cycle emissions.^23^ Without accounting for this temporal dimension, LCAs of emerging chemical production routes risk misrepresenting their true potential, leading to suboptimal policy and investment decisions.

To consider how future changes in the global economy could impact the environmental performance of chemical processes, prospective LCA has emerged as an important tool. In essence, prospective LCA leverages scenario projections to represent global supply chains under future conditions.^24^ Some recent works applying prospective LCAs to platform chemicals provide valuable insights into the performance of alternative production routes under expected climate scenarios in the future.^24,25^ Prospective assessments can indeed significantly affect the insights derived from static assessments (i.e., those conducted considering LCA data of existing economic systems). For instance, Medrano-García et al.^26^ showed that while alternative vinyl chloride monomer (VCM) synthesis routes from ethane are disadvantageous in a 2020 scenario, they hold the potential to outperform the business-as-usual (BAU) ethylene production in a 2050 scenario. Other prospective LCAs include the work by Boyce et al.,^25^ who studied the decarbonization of ammonia production, projecting up to a 70% reduction in climate change impacts, but noting that complete decarbonization of this sector is unlikely by 2050. Moreover, Nabera et al.^24^ analyzed regionalized performance of hydrogen, ammonia, and methanol, projecting life cycle greenhouse gas (GHG) emissions reductions up to 90% by 2050.

In this work, we compare the economic and environmental performance of propanol production via the fossil-based and alternative low-carbon production processes. More specifically, we analyze various propanol production scenarios within the conventional hydroformylation-hydrogenation process by varying the feedstock supply routes (using captured CO_2_, electrolytic hydrogen, and biogas). By combining process simulation, TEA, and prospective LCA, we provide a comprehensive analysis of the potential advantages and trade-offs associated with each production route currently and in the future. As mentioned previously, the main novelty of the work lies in a comprehensive economic and environmental evaluation of the direct biogas-to-syngas (dry reforming) option for propanol production. Overall, we find that the low-carbon route based on biogas could significantly reduce both the climate change impact and the production cost of propanol, with future decarbonization efforts further enhancing the environmental appeal of these alternative routes.

## Methods

### Propanol Production Routes

An overview of the propanol production routes assessed in this study are provided in Figure 1. Note that we use the term “route” to distinguish between the various pathways studied in this work, with all routes undergoing the hydroformylation-hydrogenation process but differing in the feedstocks used. Therefore, these routes are not different reaction pathways to propanol, but different routes to produce the feedstocks required in the hydroformylation-hydrogenation process for propanol production. The considered process configurations include the conventional fossil-based route and four low-carbon pathways using captured CO_2_, electrolytic hydrogen, and biogas as feedstocks. In comparison to the BAU fossil-based route (natural gas reforming resulting in syngas with a 1:1 ratio of CO:H_2_), two key modifications were considered for the low-carbon alternatives, i.e., the use of (i) CO_2_-derived syngas obtained via the RWGS reaction using captured CO_2_ from DAC and electrolytic hydrogen,^27^ and (ii) biogas-based syngas obtained via dry reforming of the methane^28^ and CO_2_ present in biogas (57 mol % methane and 43 mol % CO_2_).^8^ DAC CO_2_ was modeled using a high-temperature liquid-solvent DAC process based on KOH absorption with Ca-looping regeneration.^29^ The syngas obtained from these alternative sources was then subjected to water removal and monoethanolamine (MEA) absorption, resulting in a 1:1 ratio of CO:H_2_. Both syngas streams were used for subsequent hydroformylation with either fossil or green ethylene, depending on the scenario.^12^ Green ethylene is obtained via the MTO route, with the methanol being produced from DAC CO_2_ and electrolytic hydrogen. The following sections describe the economic and environmental assessment methodology in more detail, with additional information provided in the Supporting Information (SI). The following scenarios are analyzed in our study:

- Fossil scenario: options (a) and (i) in Figure 1
- DAC CO_2_ and electrolytic H_2_ + fossil ethylene: options (b) and (i) in Figure 1
- Biogas + fossil ethylene: options (c) and (i) in Figure 1
- DAC CO_2_ and electrolytic H_2_ + green ethylene: options (b) and (ii) in Figure 1
- Biogas + green ethylene: options (c) and (ii) in Figure 1

**Figure 1:**
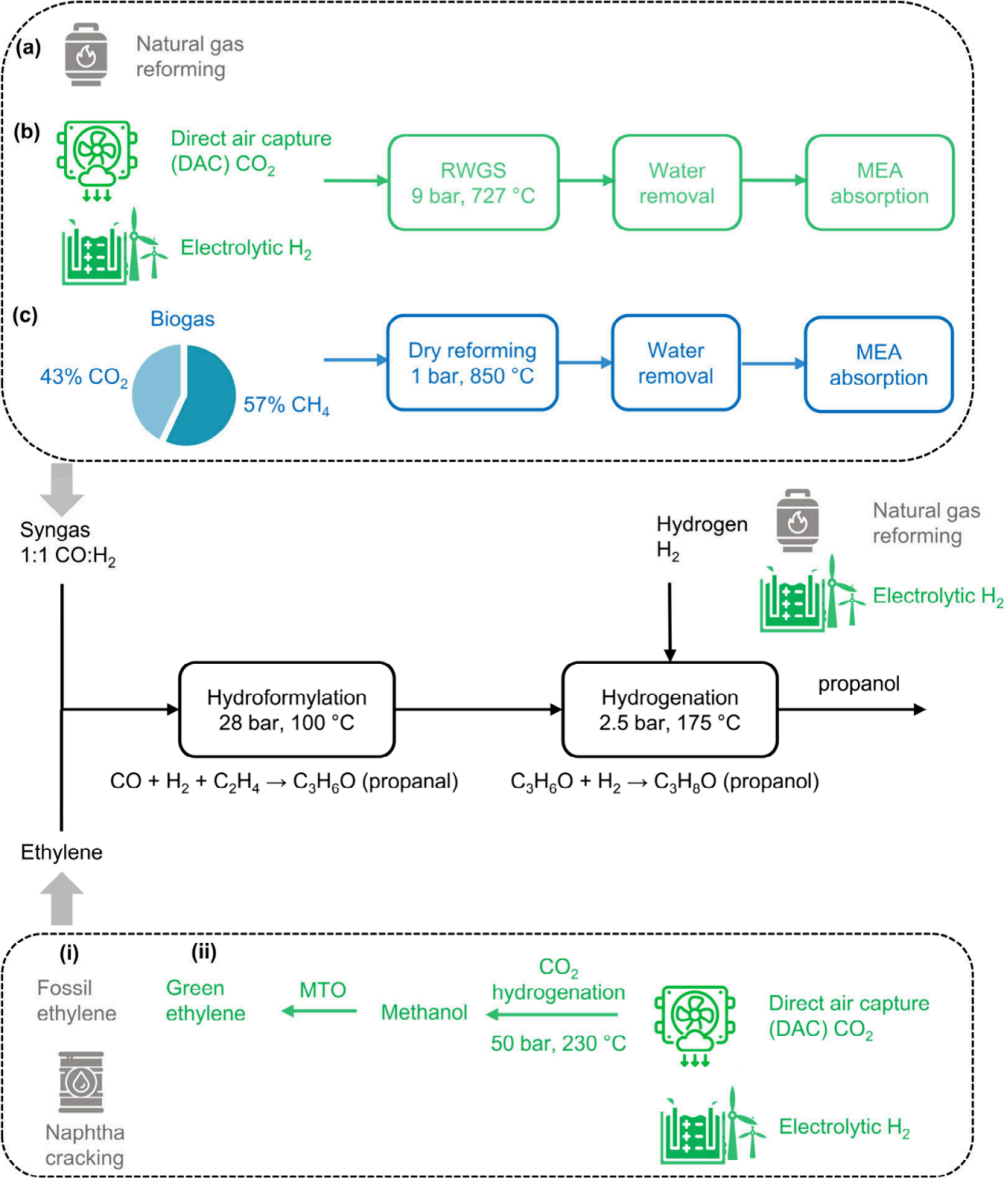
Schematic overview of propanol production routes using syngas and ethylene as precursors. Hydroformylation of ethylene with syngas yields propanal, which undergoes hydrogenation (using fossil or electrolytic hydrogen) to form propanol. Syngas production routes: (a) Conventional syngas and hydrogen are obtained from natural gas reforming. (b) Alternative syngas production via direct air capture (DAC) of CO_2_ combined with electrolytic hydrogen, via RWGS. (c) Biogas-based syngas production via dry reforming of CO_2_ and methane. Ethylene production routes: (i) Fossil-based ethylene from naphtha cracking. (ii) Green ethylene via CO_2_ hydrogenation to methanol and subsequent conversion via methanol-to-olefins (MTO). A 2 × 2 design is considered for the low-carbon cases, i.e., (b) + (i), (c) + (i), (b) + (ii), and (c) + (ii), and these alternatives are compared with the fossil scenario, i.e., (a) + (i). Note that electrolytic hydrogen is used in all low-carbon scenarios, while fossil hydrogen is used only in the fossil scenario.

### Process Simulation

The process simulations for propanol production were developed using Aspen Plus v12.1, employing the Peng–Robinson thermodynamic property package in all process units, except for the MEA absorption and regeneration section, where the Amines property package was used instead. The flowsheets were designed for an annual production rate of 100 kt yr^–1^, producing 99.5% pure propanol on a mass basis. A capacity of 100 kt yr^–1^ was chosen as a representative midscale plant size and as a standardized basis for equipment sizing and TEA/LCA comparisons across scenarios. Heat integration was performed for all assessed process configurations using Aspen Energy Analyzer v12.1, and the resulting net heating and cooling utility demands after heat recovery were used in the TEA and LCA.

The biogas route consists of using biogas (along with DAC CO_2_, essential to eventually produce syngas with a 1:1 ratio of CO:H_2_) as feedstock, undergoing dry reforming (at 850 °C, 1 bar) to produce syngas. Subsequently, water removal, and MEA absorption for CO_2_ removal result in syngas with the required CO:H_2_ ratio of 1:1. Next, the produced syngas is combined with ethylene (either fossil or green) to produce propanal (C_3_H_6_O) via the hydroformylation reaction (at 100 °C, 28 bar) shown below:

$$\mathrm{CO}+H_{2}+C_{2}H_{4}\to C_{3}H_{6}O\left(\mathrm{propanal}\right)$$

The produced propanal then undergoes hydrogenation (at 175 °C, 2.5 bar) using hydrogen obtained from natural gas reforming or electrolysis to produce propanol, as shown below:

$$C_{3}H_{6}O+H_{2}\to C_{3}H_{8}O\left(\mathrm{propanol}\right)$$

The other route utilizing DAC CO_2_ and electrolytic hydrogen is analogous to the biogas route in all process steps starting from syngas treatment (using water removal and MEA absorption). It differs only in the feedstock and its processing step, i.e., DAC CO_2_ and electrolytic hydrogen undergo the RWGS reaction (at 727 °C, 9 bar) to produce syngas. Data for green ethylene were extracted from Ioannou et al.^19^ based on the MTO process, with methanol itself derived from DAC CO_2_ and electrolytic hydrogen.^19^ Mass and energy flows for each production route were extracted from the simulations and used as inputs for the TEA and LCA. The complete mass and energy balances for each process are summarized in Table S1 of the SI. Further details on the process modeling approach are provided in Section 1 of the SI. Detailed flowsheets simulated in Aspen Plus are shown in Figure S1a and Figure S1b (for syngas production using the biogas- and DAC CO_2_-based routes, respectively), and Figure S2 for propanol production using syngas and ethylene. Detailed stream tables for these flowsheets are provided in Tables S2–S4, while the corresponding utility energy consumption (power and thermal duties) is reported in Tables S5–S6 (with heater and cooler duties reported without heat integration).

### Techno-Economic Analysis

The TEA was conducted using data obtained from Aspen Plus simulations, including equipment sizing, materials flows, and energy requirements. The total production cost (USD kg^–1^ propanol, reported in 2023 USD) was estimated as the sum of capital expenditures (CAPEX) and operational expenditures (OPEX). OPEX was determined considering the costs of feedstock (e.g., ethylene, biogas, hydrogen) and utilities (electricity, heating, and cooling). Feedstock and utility prices for the year 2023 were taken from literature as shown in Table S7 of the SI. CAPEX was estimated using cost correlations provided by Sinnott and Towler^30^ and process unit sizes derived from simulation results. The obtained CAPEX was annualized using an annual capital charge ratio (ACCR) of 0.13, calculated from an interest rate of 12% and a project lifetime of 25 years, in accordance with Sinnott and Towler.^30^ When necessary, historical values were adjusted using the average chemical engineering plant cost index (CEPCI). A full description of the TEA methodology is provided in Section 2 of the SI.

### Life Cycle Assessment

Environmental impacts were evaluated using an attributional cradle-to-gate LCA in accordance with ISO 14040/14044 standards.^31,32^ The functional unit was defined as 1 kg of propanol at factory gate. The system boundary encompasses all processes from feedstock acquisition to the production of 99.5 wt % pure propanol. Alternatively, an upstream functional unit (such as an equimolar mixture of syngas and ethylene) could have been defined, as the hydroformylation and hydrogenation processes are present in all studied scenarios. However, as the intended objective of this analysis is n-propanol defossilization and an equimolar syngas and ethylene mixture is not a typically used market commodity, we retain the hydroformylation-hydrogenation section, and choose 1 kg of propanol at the factory gate as the functional unit. The end-use stage is omitted because it is assumed to be identical across all production routes and, therefore, adds no discriminatory power to the comparative analysis. Foreground data (i.e., data for processes directly involved in propanol production) were obtained directly from process simulations and literature sources,^8,19^ while background data (i.e., other sectors such as power generation) were sourced from the ecoinvent v3.10 database (cutoff system model).^33^ Climate change impacts were quantified using the IPCC 2021 global warming potentials (GWPs) over a 100-year time horizon.^34^ Additionally, the ReCiPe 2016 v1.03 method was used to evaluate end point impacts on human health, ecosystem quality, and natural resources.^35^ Further information regarding the corresponding life cycle inventories (LCIs) for all the low-carbon routes are provided in Table S8 of the SI.

To evaluate future environmental performance, a prospective LCA was carried out for the year 2050 using the *premise* v2.1.3 framework.^23^ This approach adjusts background data based on scenarios derived from integrated assessment models (IAMs),^36^ capturing the expected decarbonization of key industrial sectors, such as energy, transportation, fuels supply, and heavy industry. More specifically, we used a scenario from the IMAGE IAM corresponding to a trajectory aligned with the shared socioeconomic pathway SSP2 (‘middle-of-the-road’) and the representative concentration pathway RCP2.6, corresponding to a global mean surface temperature increase limited to 2 °C. The full description of the methodology is presented in Section 3 of the SI. For the uncertainty assessment, we use the ecoinvent Pedigree matrix (containing uncertainty information on the LCI parameters) to randomly generate 500 different backgrounds via Monte Carlo sampling.^37,38^ The Pedigree matrix considers diverse quantitative criteria, including geographical and temporal aspects, to model the uncertainty distribution of LCI parameters in the ecoinvent database. Further assumptions and limitations of this study are detailed in Section 4 of the SI.

## Results and Discussion

### Economic Assessment

As shown in Figure 2a, we compare the production costs (in USD kg^–1^) of the BAU fossil process with the alternative production routes. In economic terms, we observe that the fossil process (1.3 USD kg^–1^) is outperformed only by the biogas-based alternative route (30% lower cost, i.e., 0.9 USD kg^–1^): the one utilizing the feedstock biogas (for syngas production), electrolytic hydrogen (for hydrogenation), and fossil ethylene (the combination of options (c) and (i) for syngas and ethylene, respectively, in Figure 1). The main contributors to the cost of this route are the feedstock: fossil ethylene (45%), electrolytic hydrogen (22%), and biogas (13%). CAPEX contributes 10%, followed by the heating utility (4%). The remaining components together contribute less than 5% to the production cost. Although the production process is the same as the fossil route, the key reason for the lower production cost of the biogas-based route is the cheaper feedstock (biogas at 0.3 USD kg^–1^, versus natural gas at 0.5 USD kg^–1^). This route is followed by the one using CO_2_ and electrolytic hydrogen (for syngas production), electrolytic hydrogen (for hydrogenation), and fossil ethylene (options (b) and (i) in Figure 1), with a cost of 1.5 USD kg^–1^ (11% more expensive than the fossil route). Similar to the cheapest option, the feedstock dominates the total cost (88%; the increased use of more expensive electrolytic hydrogen and DAC CO_2_ negatively affects the economics of this route), followed by CAPEX and heating utility (about 6%).

**Figure 2:**
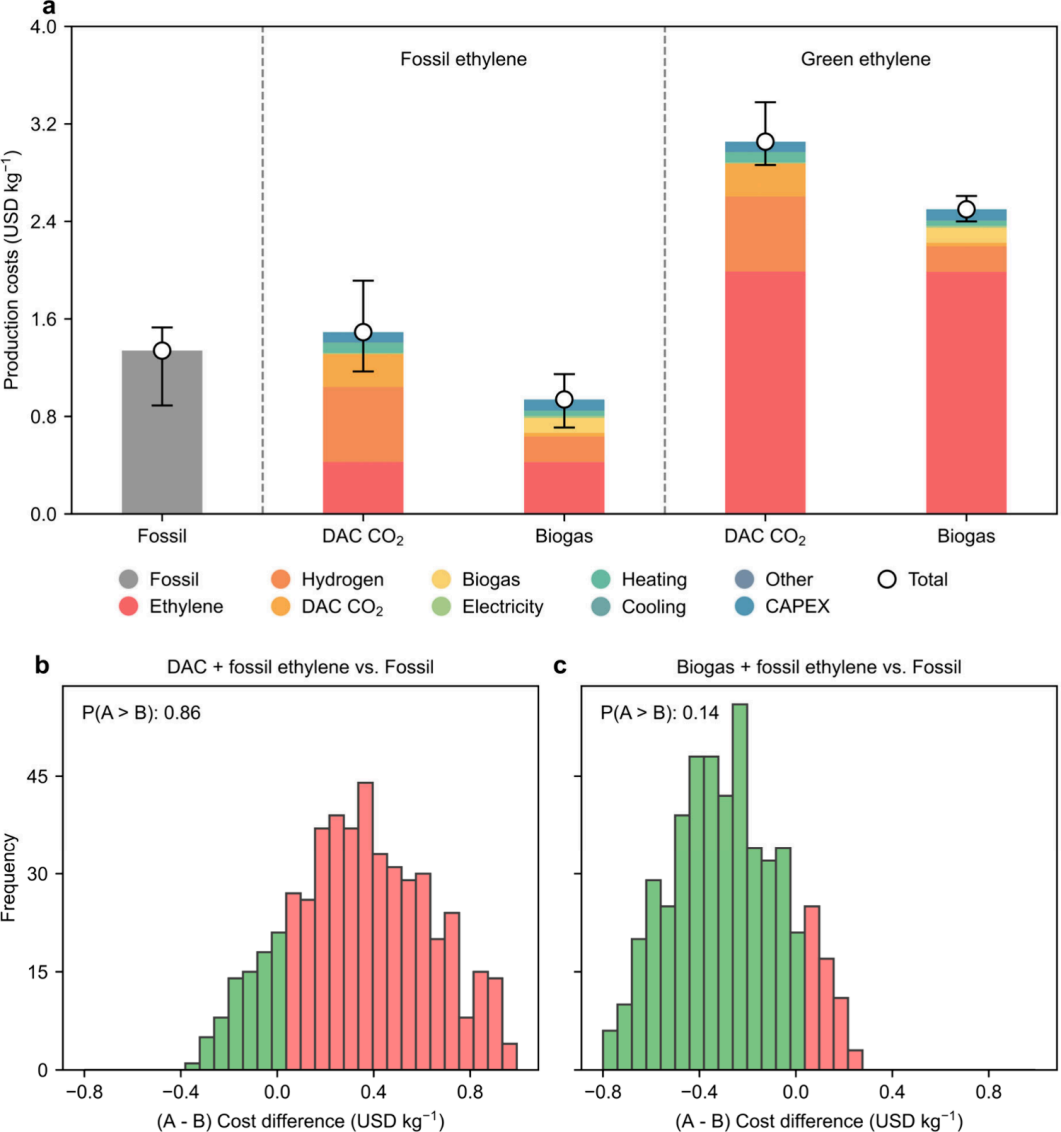
(a) Total production cost breakdown for all propanol production scenarios. The uncertainty bars show the best- and worst-case scenarios, calculated as described in section 2 of the SI. (b) Monte Carlo uncertainty analysis of the economic assessment of the route utilizing DAC CO_2_ and fossil ethylene (A) and the fossil route (B). (c) Monte Carlo uncertainty analysis of the economic assessment of the route utilizing biogas and fossil ethylene (A) and the fossil route (B). The feedstock prices are varied assuming a uniform distribution. P­(A > B) denotes the probability of the fossil route economically outperforming the alternative, low-carbon routes.

The other two routes, differing only in the ethylene source (replacing fossil with green ethylene), show a drastic increase in cost: 166% and 105% compared to the analogous routes using fossil ethylene along with biogas and DAC CO_2_, respectively (these routes are thus 87% and 128% more expensive than the fossil route). This result stems from the increase in the cost of ethylene (from a fossil ethylene cost of 0.9 USD kg^–1^ to a green ethylene cost of 4.2 USD kg^–1^). The other contributors to the production cost remain unchanged. Figure S3 presents the overall CAPEX breakdown for both the biogas and DAC CO_2_-based routes.

Therefore, the alternative route utilizing biogas, electrolytic hydrogen, and fossil ethylene has the potential to be economically competitive. Notably, this conclusion remains largely robust under both best- and worst-case scenarios of feedstock prices. As shown in Figure 2b and Figure 2c, we also performed a Monte Carlo uncertainty analysis by varying the feedstock prices of the two best-performing alternative routes and the fossil route. Comparing with the fossil route, we observe that, the cheapest route, i.e., the route utilizing biogas and fossil ethylene has a low (14%) probability of being more expensive, while the route utilizing DAC CO_2_ and fossil ethylene has a large (85%) probability of being more expensive. This highlights the significance of the biogas-based route as a viable and potentially cost-effective route, even under uncertain economic conditions. These results also indicate that, while alternative syngas production routes can be already economically viable, replacing fossil ethylene remains substantially costlier. This is primarily due to the more expensive low-carbon ethylene options, which rely on feedstocks such as waste plastics, or the more expensive electrolytic hydrogen and CO_2_ from DAC.

Several TEA studies have assessed low-carbon propanol pathways, and our results are broadly consistent with these works once differences in key assumptions are considered. For example, Vo et al.^12^ reported production costs as low as 0.9 USD kg^–1^ for CO_2_-based propanol routes; however, their analysis assumes an ethylene price of ∼ 0.1 USD kg^–1^, which is substantially lower than the range typically reported in the literature. In our case, using a fossil ethylene price of 0.9 USD kg^–1^, the DAC CO_2_ + fossil ethylene scenario remains slightly more expensive than the fossil route (1.5 versus 1.3 USD kg^–1^). Adopting an ethylene price similar to that of Vo et al.,^12^ the corresponding cost in our study decreases to 1.1 USD kg^–1^ (i.e., within 20% of their reported value), indicating that the main difference is the assumed ethylene price. In addition, other studies have assessed direct CO_2_ hydrogenation to propanol, and typically report costs that are 2–5 times higher than the fossil route.^21^

### Environmental Assessment

In terms of climate change impacts (in kg CO_2_e kg^–1^) shown in Figure 3, we observe that currently, all alternative production routes outperform the fossil process (4.4 kg CO_2_e kg^–1^). Specifically, the route utilizing green ethylene along with biogas performs best (−0.5 kg CO_2_e kg^–1^), followed by that using green ethylene and DAC CO_2_ (−0.4 kg CO_2_e kg^–1^). For both these routes, the cradle-to-gate negative impacts originate from two sources: (i) the use of biogas and DAC CO_2_, which results in net CO_2_ removal from the atmosphere (on a cradle-to-gate basis), and (ii) green ethylene, which in turn is obtained from methanol produced via DAC CO_2_ and low-carbon electrolytic hydrogen. Notably, in our analysis, we omit the end-use phase, as we assume it remains consistent across all cases regardless of the production route, and therefore would add no discriminatory power to the analysis. However, as shown in Figure 3, we evaluate cradle-to-grave impacts assuming that the embedded carbon contained in propanol is fully released as CO_2_ emissions during the use phase, providing a pessimistic estimate since not all carbon content may be emitted during its lifetime.

**Figure 3:**
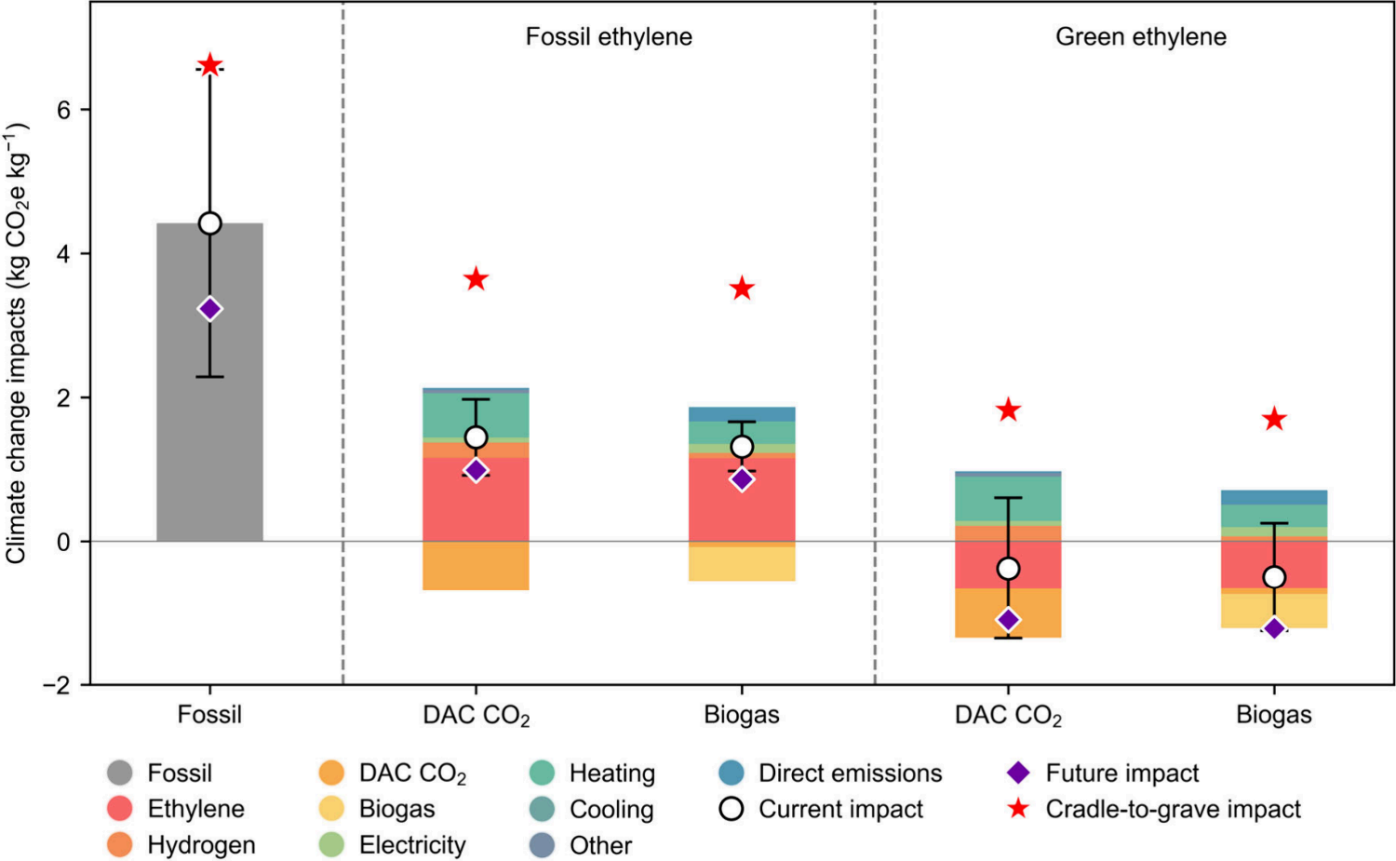
Breakdown of climate change impacts for the assessed propanol production scenarios. The uncertainty bars represent the best- and worst-case scenarios, calculated using Monte Carlo simulations, as described in more detail in section 3 of the SI. The red star indicates the current cradle-to-grave impact, assuming the release of embedded carbon as CO_2_ emissions.

Most of the impact of the biogas-based route originates from heating utility (43%), direct emissions (28%), electricity (18%), and hydrogen (10%), while for the DAC CO_2_-based route, the main contributors are heating utility (63%), hydrogen (22%), electricity (7%), and MEA required for CO_2_ absorption (5%). The other two routes utilizing biogas and DAC CO_2_ coupled with fossil ethylene perform better than the fossil process (70% and 63% lower impacts, respectively), yet they show overall positive impacts (1.4 and 1.3 kg CO_2_e kg^–1^, respectively) due to large GHG emissions associated with fossil ethylene. In these two routes, the net removal of CO_2_ from the atmosphere originating from biogas and DAC CO_2_ is largely counterbalanced by the emissions generated by fossil ethylene, hydrogen, utilities, and direct emissions. Here, fossil ethylene is the main contributor (biogas route: 62%; DAC CO_2_ route: 54%), followed by the utilities (biogas route: 24%; DAC CO_2_ route: 35%).

Additionally, we performed an uncertainty assessment using Monte Carlo simulations, resulting in best- and worst-case scenarios. Analogous to the economic assessment, it is observed that the trends described previously remain largely unchanged.

The prospective scenarios shown in Figure 3 for the year 2050 indicate significant impact reductions across scenarios, ranging from 27% for the fossil route to 193% for the DAC CO_2_- and green ethylene-based route. The main reason for this observation is the drastically reduced impact for the global electricity mix by 2050. However, the trend described for the current conditions remains unchanged: the fossil process is expected to perform the worst (3.2 kg CO_2_e kg^–1^), while the green ethylene-based route with biogas would continue to be the best-performing option (−1.2 kg CO_2_e kg^–1^), followed by that using green ethylene and DAC CO_2_ (−1.1 kg CO_2_e kg^–1^). The routes based on fossil ethylene are expected to continue showing overall positive impacts in the future (biogas-based route: 0.9 kg CO_2_e kg^–1^; DAC CO_2_-based route: 1.0 kg CO_2_e kg^–1^), but still perform better than the fossil route. It should be noted that uncertainty analysis has not been applied to the LCIs predicted for the prospective scenario because *premise*-generated inventories lack the necessary information to model the underlying probability distributions.^39^ The breakdown of climate change impacts in the 2050 future scenario is presented in Section 5, Figure S4 of the SI.

Additionally, our results help demonstrate why biogas-based propanol routes reported in the literature can lead to markedly different outcomes depending on how biogas is processed. For example, Motte et al.^11^ assessed a route based on upgrading biogas to biomethane prior to propanol production and reported cradle-to-gate climate change impacts of 6.0 kg CO_2_e kg^–1^ propanol, i.e., higher than our fossil reference (4.4 kg CO_2_e kg^–1^). In contrast, in our work, the direct biogas-to-syngas (dry reforming) + fossil ethylene scenario achieves 1.3 kg CO_2_e kg^–1^, corresponding to a ca. 70% reduction relative to the fossil route and 80% lower impacts than the upgrading-based biogas route. This improved performance primarily results from removing the upgrading step, thereby avoiding its additional energy demand and potential methane leakage burdens, and from higher carbon utilization, since both CO_2_ and methane in biogas are converted to syngas and subsequently incorporated into propanol rather than being separated and released.

We next compare the environmental performance of the economically best-performing process (i.e., the biogas-based route utilizing fossil ethylene) with the fossil route for impact categories other than climate change, using uncertainty assessment of the background data. The main aim of this analysis is to ascertain the occurrence of burden shifting (i.e., improvement in one impact category at the expense of worsening others).^40^ Accordingly, we used the ReCiPe 2016 v1.03 method^35^ to assess the three end point categories, i.e., human health (in disability-adjusted life years, DALY kg^–1^), ecosystem quality (in species·yr kg^–1^), and natural resources (in USD 2013 kg^–1^). As shown in Figure 4a, the biogas-based route outperforms the fossil route across all three end points, with the feedstock (ethylene, followed by biogas) and heating utility emerging as the main contributors for the biogas-based route. Similar results for the other production routes are presented in Section 6 of the SI, with Figure S5 showing human health, Figure S6 showing ecosystem quality, and Figure S7 showing the natural resources impact categories. Further, an uncertainty analysis was conducted by calculating the end point impacts of the biogas-based route utilizing fossil ethylene and the fossil route for 500 different backgrounds, with the paired comparison shown in Figure 4b. We find that the biogas-based route utilizing fossil ethylene improves climate change impact without causing significant burden shifting across other environmental categories. In particular, its performance also improves outcomes for human health, ecosystem quality, and natural resource use. Section 7 of the SI presents comparable results for the uncertainty assessment of the DAC CO_2_-based route using fossil ethylene, the DAC CO_2_-based route using green ethylene, and the biogas-based route using green ethylene, as shown in Figures S8, S9, and S10, respectively. Here, we find that the highest probability of burden shifting occurs when green ethylene is used, particularly affecting the human health impact category.

**Figure 4:**
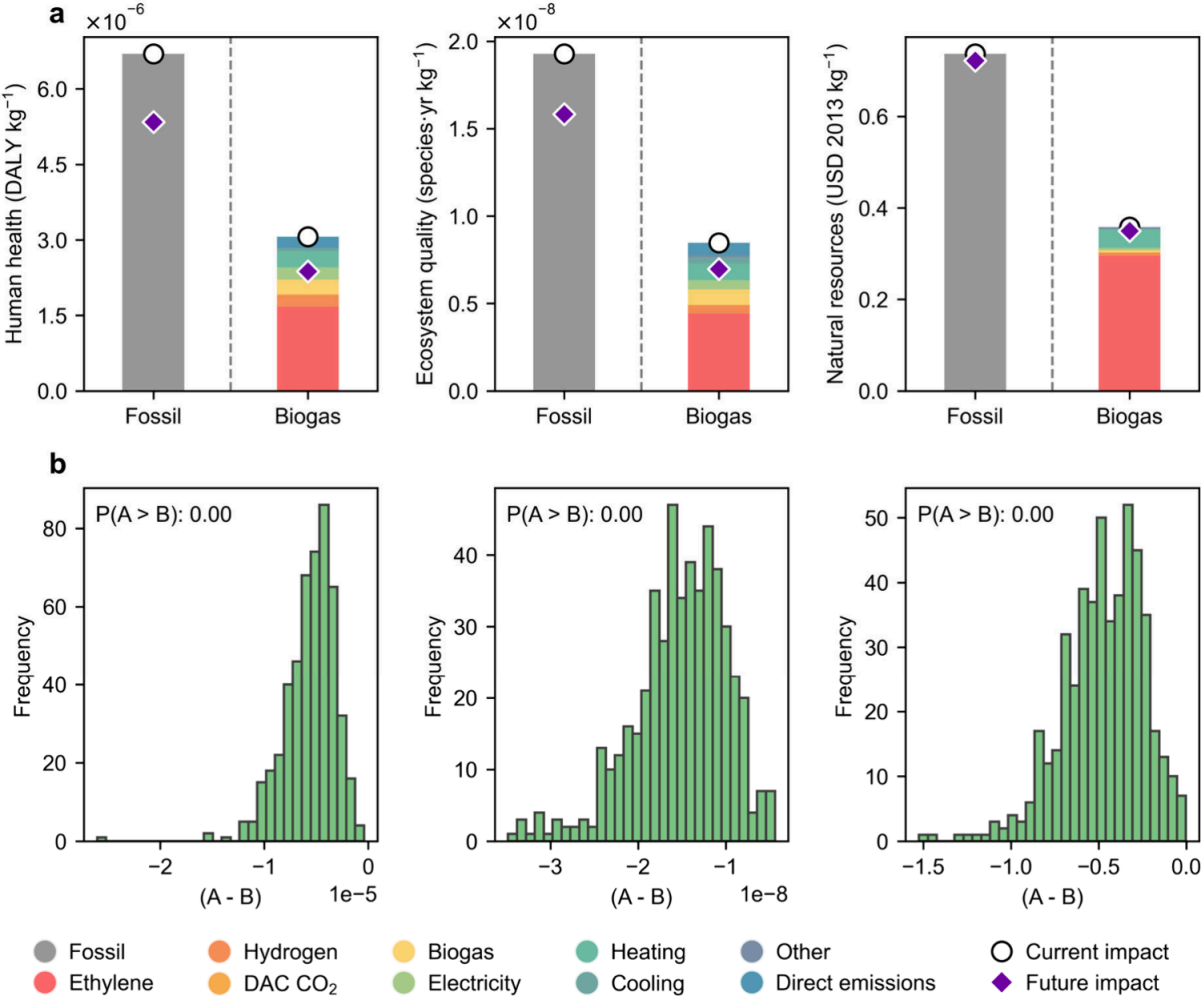
(a) Impact results per kg of propanol for the fossil-based business-as-usual (BAU) route and biogas-based route utlizing fossil ethylene, calculated across the three end point categories of the ReCiPe 2016 v1.03 method. (b) Probability of burden-shifting (A > B) for propanol production technologies across the three end point categories. Here, A represents the biogas-based route utilizing fossil ethylene, and B represents the fossil-based BAU route. Green bars indicate cases in which the environmental footprint of the fossil process is lower than that of the biogas-based process, whereas red bars indicate cases in which the environmental footprint of the fossil scenario is higher. Burden shifting is considered to occur when P­(A > B) ≥ 0.75, while no burden shifting is observed when P­(A > B) < 0.25.

### A Decarbonization Roadmap for Propanol Production

Considering the current global annual propanol production of 4 Mt and the estimated cradle-to-gate climate change impacts of the fossil process of 4.4 kg CO_2_e kg^–1^, current annual GHG emissions amount to approximately 17.7 Mt CO_2_e (similar to annual GHG emissions of countries such as Costa Rica and Mauritania).^41^ Moreover, assuming a projected demand growth rate of 5% per year, global propanol demand could reach about 13.5 Mt by 2050, corresponding to 43.7 Mt CO_2_e if current production practices remain unchanged. In view of global ambitions to achieve net-zero GHG emissions in the second half of the century, these emissions represent a significant mitigation challenge. Building on our findings, we propose a roadmap to decarbonize propanol production.

In the short term, our results indicate that substituting natural gas with biogas for syngas production, in combination with fossil ethylene, represents a win-win scenario: it not only outperforms the fossil route economically but also across all environmental impact categories analyzed (i.e., climate change, human health, ecosystem quality, and natural resources). Incorporating biogas into existing operations would enable propanol producers to reduce GHG emissions cost-effectively by leveraging existing infrastructure and high-TRL technologies, while simultaneously reducing exposure to volatile natural gas prices.^8^ This would be particularly relevant for facilities located in regions that rely heavily on natural gas imports, such as Europe. However, integrating biogas into existing propanol production facilities presents significant logistical challenges. Due to its relatively low value, transporting biogas over long distances is not feasible, meaning production and use must be colocated. Anaerobic digestion facilities treating biodegradable waste would need to be situated near propanol production plants. While this is technically feasible, ensuring a stable supply of waste feedstock near these facilities is challenging. Consequently, successful integration depends on the availability of a reliable local source of waste feedstock. Future work could focus on spatially explicit assessments that combine the locations of existing propanol plants with maps of organic waste availability.

In the longer term, propanol production must transition toward net-zero GHG emissions. Our results demonstrate that achieving cradle-to-gate negative emissions is technically feasible even under current conditions; however, this would require switching to green ethylene produced from DAC CO_2_ and electrolytic hydrogen. Such a shift would substantially increase production costs, largely due to the large amount of energy required. Consequently, securing a reliable supply of low-carbon ethylene may become the main bottleneck for propanol producers on their route to net-zero. The use of waste plastics in a circular production route (waste plastics-to-methanol-to-ethylene) can be a viable high-TRL option, resulting in about 26% lower climate change impact at an economic premium of only 23%.^39^ Thus, in the future, precursors for propanol production could be supplied by integrated waste management facilities that generate biogas through anaerobic digestion of organic waste and recover plastic waste for conversion into ethylene.

Using biogas to produce syngas for propanol production represents a high-value application compared with its more common uses, such as electricity and heat generation and upgrading to biomethane. For example, biomethane production could generate between 0.3 and 0.6 USD per m^3^ of biogas, assuming a biomethane market price of 59–106 USD MWh^–1^ and a conversion rate of 0.58 m^3^ biomethane per m^3^ of biogas.^8,18^ In contrast, propanol market prices range from 0.9 to 1.5 USD kg^–1^, and 2.9 kg of propanol can be produced per m^3^ biogas, yielding 2.6 to 4.4 USD per m^3^ of biogas. From a climate change mitigation perspective, propanol may also offer a more efficient use of biogas. For example, future electricity generation from biogas is likely to provide limited benefits due to the growing share of wind and solar power. Meanwhile, there are limited cost-effective alternatives to produce low-carbon syngas for propanol production, as demonstrated in this study. As biogas is a limited resource constrained by the availability of organic waste, its use should be prioritized for applications where it delivers the greatest benefits. In this work, we demonstrate its potential for decarbonizing a challenging commodity such as propanol, whereas previous studies have explored biogas utilization for ammonia^8^ or hydrogen^42^ production. Future research could build upon our findings and data to assess the overall availability of biogas alongside all possible end uses, thereby identifying the most economically- and environmentally effective utilization pathways.

Propanol production has a substantial impact on the carbon footprint of downstream sectors, such as the cosmetics and pharmaceutical industries. Currently, propanol contributes 13–77% of the carbon footprint of a range of products in these sectors, including a 45% contribution to the carbon footprint of propyl acetate (used as a solvent), 73–77% for propyl amine (used in the synthesis of pharmaceuticals), and 14% for prochloraz (used in agriculture for crop protection) (see Section 3 and Table S9 of the SI). Consequently, a 70% reduction in GHG emissions from propanol production using syngas from biogas could reduce its contribution to 4–24% in the said final products. These estimates highlight that decarbonizing propanol has far-reaching implications across global supply chains. In the context of increasingly stringent GHG emissions regulations (e.g., an increasing number of pharmaceutical companies are committing to reach net-zero by 2050),^43^ decarbonizing propanol emerges as a critical mitigation strategy.

## Conclusions

In this work, we evaluated the economic and environmental performance of various propanol production routes, including fossil-based routes as well as alternative approaches utilizing captured CO_2_, electrolytic hydrogen, and biogas. The economic assessment was carried out using techno-economic analysis (TEA), while life cycle assessments (LCAs) were conducted under both current and prospective scenarios to evaluate how anticipated trends could influence environmental performance.

We observed that the biogas-based route, i.e., the process utilizing biogas (for syngas production), electrolytic hydrogen (for hydrogenation), and fossil ethylene is a win-win scenario: this process outperforms the fossil route economically as well as environmentally (production cost of 0.9 USD kg^–1^ and current climate change impact of 1.4 kg CO_2_e kg^–1^, i.e., 30 and 70% lower than the analogous fossil route metrics, respectively). Extending the analysis to other impact categories, such as human health, ecosystem quality, and natural resources, shows no burden-shifting for the biogas-based route utilizing fossil ethylene, further emphasizing its potential as a promising alternative. The key reasons for the improved economic and environmental performance are the use of a cheaper feedstock (biogas) and the use of biogenic CO_2_, respectively.

On the other hand, while the routes utilizing green ethylene along with both biogas and DAC CO_2_ outperformed all other routes in the environmental assessment, the high cost of green ethylene negatively impacted their economic performance. Furthermore, a prospective LCA for the year 2050 highlighted the dominant role of the future grid electricity decarbonization in drastically reducing climate change impacts across all production routes. This prospective LCA shows that the win-win route described earlier, i.e., the biogas-based route utilizing fossil ethylene, is expected to continue performing better than the fossil route in the future (73% lower impact of 0.9 kg CO_2_e kg^–1^).

Overall, these results underscore the potential of alternative routes using biogas as a viable and cost-effective approach for low-carbon propanol production, paving the way for future investigations into other platform chemicals.

## Supplementary Material



## Data Availability

The data presented in the figures of this paper are publicly available via Zenodo (10.5281/zenodo.17777161). The background LCI data sets used in this study are available in the ecoinvent v3.10 (cutoff system model) database under the accessible link (https://ecoinvent.org).

## Notes

Process modeling methodology (section 1), techno-economic analysis methodology (section 2), life cycle assessment methodology (section 3), assumptions and study limitations (section 4), a breakdown of climate change impacts in 2050 (section 5), additional impact categories (section 6), and an uncertainty analysis (section 7), along with references (PDF)
