# Supplementary material for: Biogas Could Enable Cost-Effective Defossilization of n‑Propanol and Its Derivatives
Source: ACS Sustain Chem Eng. 2026 Mar 20;14(13):6365–74. doi: 10.1021/acssuschemeng.5c13160 (PMC13058886; doi:10.1021/acssuschemeng.5c13160)
Supplement: Supplementary file 1 [file sc5c13160_si_001.pdf]

Supporting Information

Biogas could enable cost-effective defossilization of n-propanol and its derivatives

Abhinandan Nabera,[†a,b] Sachin Jog,[†a] Juan D. Medrano-García,[a,b] Robert Istrate,[c] and Gonzalo Guillén-Gosálbez*[a,b]

[a] *Institute for Chemical and Bioengineering, Department of Chemistry and Applied Biosciences, ETH Zurich, Vladimir Prelog Weg 1, Zurich 8093, Switzerland.*

[b] *NCCR Catalysis, Zurich 8093, Switzerland.*

[c] *Institute of Environmental Sciences (CML), Leiden University, Einsteinweg 2, 2333 CC Leiden, The Netherlands.*

[†] Equal contribution.

* *Corresponding author. Email: gonzalo.guillen.gosalbez@chem.ethz.ch*

Table of contents

1. Process modelling methodology 2

2. Techno-economic analysis methodology 13

3. Life cycle assessment methodology 16

4. Assumptions and limitations of the study 19

5. Breakdown of climate change impacts in 2050 20

6. Additional impact categories 21

7. Uncertainty analysis 24

References 28

1. Process modelling methodology

This section provides additional details on the process modelling. Two process models have been implemented in Aspen Plus v12.1: (i) the biogas route (**Figure S1a** and **Figure S2**), and (ii) the route utilizing direct air capture (DAC) CO_2 and electrolytic hydrogen (**Figure S1b** and **Figure S2**). The annual production rate of n-propanol (referred to as propanol in the rest of the Supporting Information) is 100 kt yr^{-1} (±10%) in both plants, producing 99.5% pure propanol (mass basis). We use the Peng-Robinson thermodynamic property package in all process units, except for the monoethanolamine (MEA) absorption and regeneration section, where the Amines property package is used. We now describe the two process models in further details. Heat integration was performed for all assessed process configurations using Aspen Energy Analyzer v12.1, and the resulting net heating and cooling utility demands were used in the TEA and LCA.

Biogas route

See **Figure S1a** and **Figure S2** for the detailed flowsheets. As shown in **Figure S1a**, the fresh feed (at 25 °C, 1 bar) in this route consists of biogas (57% methane and 43% CO_2)[1] and DAC CO_2. The DAC CO_2 ensures a CH_4:CO_2 ratio of 1:1 in the fresh feed, which in turn ensures a CO:H_2 ratio of 1:1.[2] The input stream is heated to 850 °C (the reactor's operating temperature) and then sent to a Gibbs reactor, where the dry reforming reaction occurs. Dry reforming was modelled as an equilibrium (Gibbs) reactor because at 850 °C the reaction is expected to be close to thermodynamic equilibrium.[2] Dry reforming was operated at 1 bar, consistent with previous studies.[2]

The reactor outlet, consisting mainly of CO and H_2 (produced in a 1:1 ratio) along with CO_2 and water, is cooled to 25 °C and then compressed to 28 bar using multi-stage compression with inter-stage cooling. Flash units in this section remove excess water.

The next step involves CO_2 removal. The compressed syngas stream, together with a stream of fresh MEA and water (heated to 40 °C and compressed to 28 bar), is sent to the MEA absorption column (10 equilibrium stages) for CO_2 removal. This results in a syngas stream consisting mainly of CO and H_2 in a 1:1 ratio. The MEA used for CO_2 removal is regenerated in the bottom stream of a distillation column (10 equilibrium stages) and then recycled to the absorption column inlet. The distillate stream of the column, consisting mainly of water and a small amount of CO_2, is sent to a flash unit, which separates water and CO_2 (the latter being recycled to the inlet of the dry reforming reactor).

Next, as shown in **Figure S2**, the syngas is combined with ethylene (compressed to 28 bar with inter-stage cooling) and heated to 100 °C (the reactor's operating temperature) for the hydroformylation reaction. A stoichiometric reactor in Aspen Plus is used, with the yield of propanal (the main product) being 99% and that of the side product (ethane) being 1%.[3] The resulting propanal is heated to 175 °C and combined with hydrogen (175 °C, 2.5 bar) for the hydrogenation reaction. This reaction occurs in a plug flow reactor (PFR; length 19.69 m, diameter 3.94 m) to yield propanol. The kinetics were obtained from Vo *et al*.[3] The reactor outlet is then cooled to 25 °C and separated in a flash unit to yield 99.5% pure propanol (mass basis) in the liquid stream, while the vapor stream, consisting mainly of methane, carbon monoxide, and ethane, is purged. **Table S1** presents the detailed mass and energy flows of the biogas-based route.

DAC CO_2 and electrolytic hydrogen route

See **Figure S1b** and **Figure S2** for the detailed flowsheets. As shown in **Figure S1b**, the fresh feed consists of DAC CO_2 (at 25 °C, 1 bar) and electrolytic hydrogen from a proton exchange membrane (PEM) electrolyzer (at 80 °C, 30 bar). After compressing the DAC CO_2 stream to 9 bar using multi-stage compression with inter-stage cooling and expanding the electrolytic hydrogen stream to 9 bar through a turbine, the feedstock are heated to 727 °C. This is the input stream for the reverse water-gas shift (RWGS) reaction, modelled using a Gibbs reactor. RWGS was modelled as an equilibrium (Gibbs) reactor because at 727 °C the reaction is expected to be close to thermodynamic equilibrium.[4] The reactor outlet, consisting mainly of CO and H_2 in a 1:1 ratio along with CO_2 and water, is cooled to 25 °C, followed by water removal in a flash unit and subsequent compression to 28 bar. Note that while the subsequent steps for propanol production in this route are similar to those in the biogas route, the full description is provided here for completeness.

Analogous to the biogas route, the next step is CO_2 removal using MEA absorption. Therefore, the compressed syngas stream is combined with a stream of fresh MEA and water (heated to 40 °C and compressed to 28 bar) and sent to the MEA absorption column (10 equilibrium stages) for CO_2 removal. The resulting syngas stream now consists mainly of CO and H_2 in a 1:1 ratio. The MEA is regenerated in the bottom stream of a distillation column (10 equilibrium stages) and then recycled to the absorption column inlet. The distillate stream of the column, consisting mainly of water and a small amount of CO_2, is sent to a flash unit, which separates water and CO_2; the latter is recycled to the inlet of the RWGS reactor.

Subsequently, as shown in **Figure S2**, the syngas stream is heated to 100 °C (the reactor's operating temperature) and combined with ethylene (compressed to 28 bar with inter-stage cooling and heated to 100 °C) for the hydroformylation reaction. A stoichiometric reactor in Aspen Plus is used, with a propanal yield of 99%, and a side product (ethane) being 1%.[3] The resulting propanal is heated to 175 °C, and combined with hydrogen (175 °C, 2.5 bar) for the hydrogenation reaction in a PFR (length 19.69 m, diameter 3.94 m), yielding propanol. The reactor outlet is then cooled to 25 °C and separated in a flash unit to yield 99.5% pure propanol (mass basis) in the liquid stream, while the vapor stream, consisting mainly of carbon dioxide, ethane, and carbon monoxide, is purged. **Table S1** presents the detailed mass and energy flows of the DAC CO_2-based route.

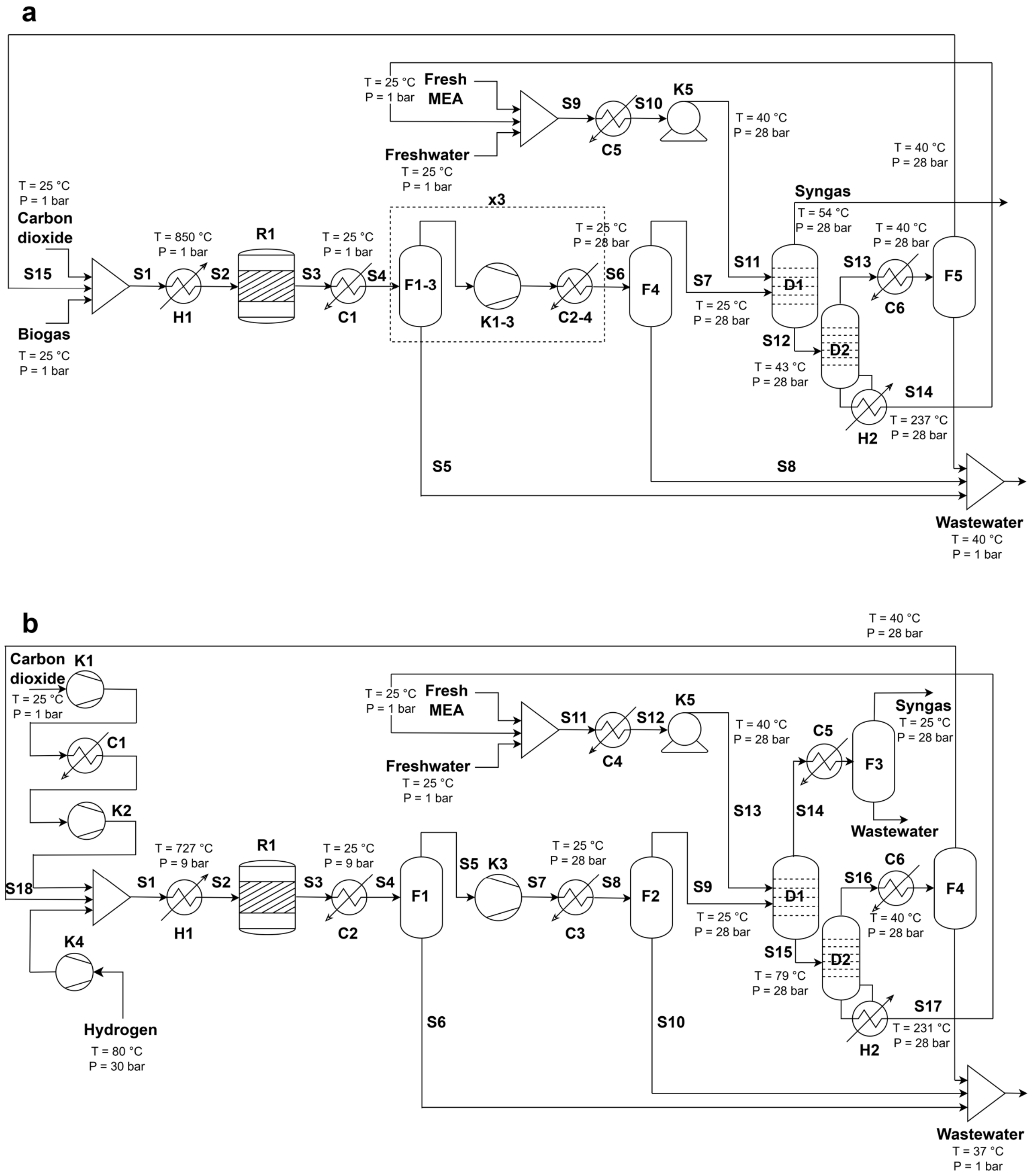

Figure S1. Process flow diagrams for syngas production via **(a)** the biogas route **(b)** DAC CO_2 and electrolytic hydrogen route.

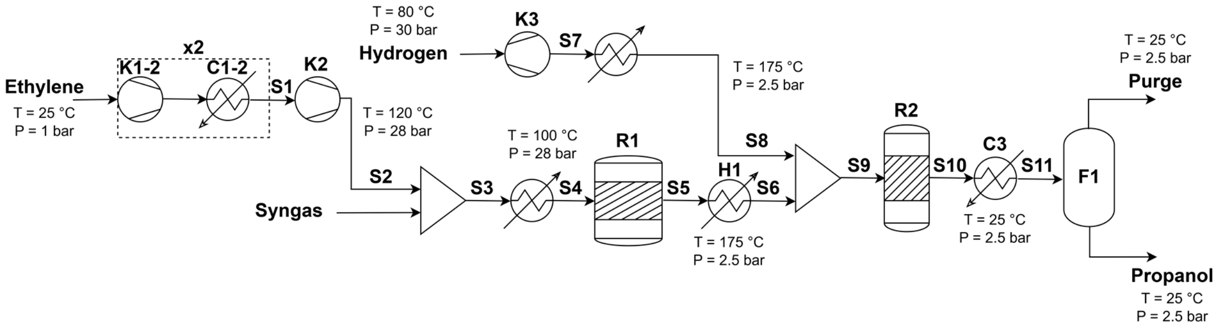

Figure S2. Process flow diagrams for propanol production via syngas and ethylene.

Table S1. Inputs and outputs of the DAC CO_2- and biogas-based production routes per kg propanol produced. For both routes, these values were extracted from the Aspen Plus v12.1 simulation (**Figures S1**, **S2**). Biogas volumes are reported in m^3 at 25 °C and 1 bar.

Stream	DAC CO_2	Biogas
**Feedstock**		
DAC CO_2 / kg	0.747	0.084
Electrolytic hydrogen / kg	0.101	0.034
Biogas / m^3	–	0.343
Ethylene / kg	0.470	0.469
**Utilities**		
Electricity / kWh	0.106	0.188
Monoethanolamine / kg	0.012	0.001
Tap water / kg	0.043	0.004
Wastewater treatment / m^3	$35 \cdot 10^{-5}$	$59 \cdot 10^{-7}$
Cooling water / MJ	10.682	4.083
Heating (natural gas) / MJ	8.613	4.334
**Emissions to air**		
Carbon monoxide / kg	0.003	0.005
Hydrogen / kg	$49 \cdot 10^{-5}$	$57 \cdot 10^{-6}$
Carbon dioxide / kg	0.008	–
Ethane / kg	0.004	0.003
Isopropanol / kg	0.001	0.001
Methane / kg	–	0.007

Table S2. Stream table for syngas generation from biogas (**Figure S1a**). Temperature, pressure, total molar flow rate, and molar composition of all process streams in the biogas-to-syngas Aspen Plus flowsheet shown in **Figure S1a** (including fresh feeds and intermediate streams). Stream numbering corresponds to the labels in **Figure S1a**.

	**Carbon dioxide**	**Biogas**	**S1**	**S2**	**S3**	**S4**	**S5**
Temperature / °C	25.0	25.0	24.8	850.0	850.0	25.0	25.0
Pressure / bar	1.0	1.0	1.0	1.0	1.0	1.0	9.0
Molar flow / kmol hr^{-1}	23.8	191.1	217.9	217.9	423.7	423.7	1.8
Composition							
CO	0.00	0.00	0.00	0.00	0.49	0.49	0.00
H_2	0.00	0.00	0.00	0.00	0.48	0.48	0.00
H_2O	0.00	0.00	0.00	0.00	0.01	0.01	1.00
CO_2	1.00	0.43	0.50	0.50	0.01	0.01	0.00
MEA	0.00	0.00	0.00	0.00	0.00	0.00	0.00
Methane	0.00	0.57	0.50	0.50	0.01	0.01	0.00
	**S6**	**S7**	**S8**	**Fresh MEA**	**Fresh water**	**S9**	**S10**
Temperature / °C	0.0	0.0	0.0	25.0	25.0	100.7	40.0
Pressure / bar	28.0	28.0	28.0	1.0	1.0	1.0	1.0
Molar flow / kmol hr^{-1}	421.9	420.8	1.1	1.6	97.3	175.0	175.0
Composition							
CO	0.50	0.50	0.00	0.00	0.00	0.00	0.00
H_2	0.48	0.48	0.00	0.00	0.00	0.00	0.00
H_2O	0.00	0.00	1.00	0.00	1.00	0.89	0.89
CO_2	0.01	0.01	0.00	0.00	0.00	0.00	0.00
MEA	0.00	0.00	0.00	1.00	0.00	0.11	0.11
Methane	0.01	0.01	0.00	0.00	0.00	0.00	0.00
	**S11**	**Syngas**	**S12**	**S13**	**S14**	**S15**	**Wastewater**
Temperature / °C	40.3	52.4	20.3	226.7	236.5	40.0	39.7
Pressure / bar	28.0	28.0	28.0	28.0	28.0	28.0	1.0
Molar flow / kmol hr^{-1}	175.0	419.8	176.0	100.0	76.0	2.8	100.1
Composition							
CO	0.00	0.50	0.00	0.00	0.00	0.00	0.00
H_2	0.00	0.48	0.00	0.00	0.00	0.00	0.00
H_2O	0.89	0.00	0.87	0.95	0.76	0.00	0.98
CO_2	0.00	0.00	0.02	0.03	0.00	1.00	0.00

MEA	0.11	0.00	0.11	0.02	0.24	0.00	0.02
Methane	0.00	0.01	0.00	0.00	0.00	0.00	0.00

Table S3. Stream table for syngas generation from DAC CO_2 (**Figure S1b**). Temperature, pressure, total molar flow rate, and molar composition of all process streams in the DAC CO_2-to-syngas Aspen Plus flowsheet shown in **Figure S1b** (including fresh feeds and intermediate streams). Stream numbering corresponds to the labels in **Figure S1b**.

	**Carbon dioxide**	**Hydrogen**	**S1**	**S2**	**S3**	**S4**	**S5**	**S6**
Temperature / °C	25.0	80.0	38.5	727.0	727.0	25.0	25.0	25.0
Pressure / bar	1.0	30.0	9.0	9.0	9.0	9.0	9.0	9.0
Molar flow / kmol hr^{-1}	212.0	418.3	929.6	929.6	929.6	929.6	722.3	207.3
Composition								
CO	0.00	0.00	0.00	0.00	0.22	0.22	0.29	0.00
H_2	0.00	1.00	0.45	0.45	0.23	0.23	0.29	0.00
H_2O	0.00	0.00	0.00	0.00	0.23	0.23	0.00	1.00
CO_2	1.00	0.00	0.55	0.55	0.32	0.32	0.42	0.00
MEA	0.00	0.00	0.00	0.00	0.00	0.00	0.00	0.00
	**S7**	**S8**	**S9**	**S10**	**Fresh MEA**	**Fresh water**	**S11**	**S12**
Temperature / °C	140.0	25.0	27.2	27.2	25.0	25.0	100.7	40.0
Pressure / bar	28.0	28.0	28.0	28.0	1.0	1.0	1.0	1.0
Molar flow / kmol hr^{-1}	722.3	722.3	721.0	1.2	12.6	700.2	3939.0	3939.0
Composition								
CO	0.29	0.29	0.29	0.00	0.00	0.00	0.00	0.00
H_2	0.29	0.29	0.29	0.00	0.00	0.00	0.00	0.00
H_2O	0.00	0.00	0.00	1.00	0.00	1.00	0.89	0.89
CO_2	0.42	0.42	0.42	0.00	0.00	0.00	0.00	0.00
MEA	0.00	0.00	0.00	0.00	1.00	0.00	0.11	0.11
	**S13**	**S14**	**S15**	**S16**	**S17**	**S18**	**Syngas**	**Wastewater**
Temperature / °C	40.2	95.4	78.8	210.1	231.2	40.0	25.0	36.7
Pressure / bar	28.0	28.0	28.0	28.0	28.0	28.0	28.0	9.0
Molar flow / kmol hr^{-1}	3939.0	433.9	4226.1	1000.0	3226.1	299.2	421.9	921.3
Composition								
CO	0.00	0.48	0.00	0.00	0.00	0.00	0.49	0.00
H_2	0.00	0.48	0.00	0.00	0.00	0.00	0.50	0.00
H_2O	0.89	0.03	0.82	0.69	0.87	0.00	0.00	0.99
CO_2	0.00	0.01	0.07	0.30	0.00	1.00	0.01	0.00
MEA	0.11	0.00	0.10	0.01	0.13	0.00	0.00	0.01

Table S4. Stream table for propanol production from syngas and ethylene (**Figure S2**). Temperature, pressure, total molar flow rate, and molar composition of all process streams in the Aspen Plus flowsheet shown in **Figure S2** (including fresh feeds and intermediate streams). Stream numbering corresponds to the labels in **Figure S2**.

	**Carbon dioxide**	**Biogas**	**S1**	**S2**	**S3**	**S4**	**S5**	**S6**
Temperature / °C	25.0	25.0	25.0	108.0	60.2	100.0	100.0	175.0
Pressure / bar	28.0	1.0	9.0	28.0	28.0	28.0	28.0	2.5
Molar flow / kmol hr^{-1}	421.9	209.2	209.2	209.2	631.1	631.1	214.8	214.8
Composition								
CO	0.49	0.00	0.00	0.00	0.33	0.33	0.01	0.01
H_2	0.50	0.00	0.00	0.00	0.33	0.33	0.00	0.00
CO_2	0.01	0.00	0.00	0.00	0.00	0.00	0.01	0.01
Ethylene	0.00	1.00	1.00	1.00	0.33	0.33	0.00	0.00
Ethane	0.00	0.00	0.00	0.00	0.00	0.00	0.01	0.01
Propanol	0.00	0.00	0.00	0.00	0.00	0.00	0.00	0.00
Propanal	0.00	0.00	0.00	0.00	0.00	0.00	0.96	0.96
	**Hydrogen**	**S7**	**S8**	**S9**	**S10**	**S11**	**Purge**	**Propanol**
Temperature / °C	80.0	-74.5	175.0	173.9	173.9	25.0	25.0	25.0
Pressure / bar	30.0	2.5	2.5	2.5	2.5	1.0	1.0	1.0
Molar flow / kmol hr^{-1}	209.5	209.5	209.5	424.3	217.2	217.2	8.5	208.7
Composition								
CO	0.00	0.00	0.00	0.00	0.01	0.01	0.17	0.00
H_2	1.00	1.00	1.00	0.50	0.01	0.01	0.36	0.00
CO_2	0.00	0.00	0.00	0.01	0.01	0.01	0.26	0.00
Ethylene	0.00	0.00	0.00	0.00	0.00	0.00	0.00	0.00
Ethane	0.00	0.00	0.00	0.00	0.01	0.01	0.18	0.00
Propanol	0.00	0.00	0.00	0.00	0.95	0.95	0.03	0.99
Propanal	0.00	0.00	0.00	0.49	0.00	0.00	0.00	0.00

Table S5. Utility energy consumption (power and thermal duties) for the biogas and DAC CO_2 flowsheets shown in **Figure S1a** and **Figure S1b**, respectively (corresponding stream specifications are reported in **Tables S2** and **S3**). Heater and cooler duties are reported without heat integration.

**Process unit**	**Biogas**	**DAC CO_2**
**Compressors**		
K1 / kW	442.89	211.76
K2 / kW	523.54	219.90
K3 / kW	543.92	762.36
**Turbines**		
K4 / kW	–	297.61
**Pumps**		
K5 / kW	5.19	80.86
**Heaters**		
H1 / kW	2660.94	7058.96
H2 / kW	1963.65	27167.1
**Coolers**		
C1 / kW	3013.92	182.88
C2 / kW	443.59	9441.6
C3 / kW	548.37	815.63
C4 / kW	652.58	16169.90
C5 / kW	348.75	405.09
C6 / kW	1365.48	10329.8

Table S6. Utility energy consumption (power and thermal duties) for propanol production from syngas and ethylene flowsheet shown in **Figure S2** (corresponding stream specifications are reported in **Table S4**). Heater and cooler duties are reported without heat integration.

**Process unit**	
**Compressors**	
K1 / kW	204.82
K2 / kW	201.72
K3 / kW	199.44
**Turbines**	
K4 / kW	258.70
**Heaters**	
H1 / kW	258.22
H2 / kW	1966.20
H3 / kW	418.74
**Coolers**	
C1 / kW	210.24
C2 / kW	218.58
C3 / kW	3493.37

2. Techno-economic analysis methodology

The process flowsheets for the biogas- and DAC CO_2-based routes (**Figures S1**, **S2**) were developed in Aspen Plus v12.1 using standard unit process models. Simulation results, including mass and energy flow and process unit sizes, were used as input for the techno-economic analysis (TEA), **Table S1** presents the detailed mass and energy balances obtained from the process simulations. The economic assessment considers both capital expenditures (CAPEX) and operation expenditures (OPEX) for the reference year 2023.

For both production routes, the CAPEX was estimated following the correlations provided by Sinnott and Towler.[5] The purchase cost of each process equipment unit was first determined from the unit sizes extracted from Aspen Plus. These purchase costs were then adjusted using installation factors to account for equipment design (f_{er} = 0.3), piping (f_p = 0.8), instrumentation and control (f_i = 0.3), electrical installations (f_{el} = 0.2), civil works (f_c = 0.3), structures and buildings (f_s = 0.2), lagging and painting (f_l = 0.1), and materials (304 SS material factor: f_m = 1.3). The sum of all purchased equipment costs corresponds to the inside battery limit (ISBL) cost. To estimate the outside battery limit (OSBL) costs (offsites), the ISBL value was multiplied by 0.3. The total of ISBL and OSBL was then further multiplied by 0.3 and 0.1 to account for design and engineering (D&E) and contingency (X) costs, respectively. The overall CAPEX was obtained as the sum of ISBL, OSBL, D&E, and X. This total investment was annualized using an annual capital charge ratio (ACCR) of 0.13, calculated from an interest rate of 12% and a project lifetime of 25 years, in accordance with Sinnott and Towler.[5] The overall breakdown of the CAPEX for both the biogas- and DAC CO_2-based routes is provided in **Figure S3**.

The OPEX was determined using the approach of Ioannou *et al.*,[6] considering the costs of feedstocks and utilities (electricity, heating, and cooling). Feedstock and utility prices used for the OPEX calculations are summarized in **Table S2**. For the biogas and DAC CO_2 routes, these include the costs of biogas, captured CO_2, electrolytic hydrogen, and utilities.

For the fossil propanol,[7] fossil ethylene,[8] and methanol-to-olefins (MTO)-derived green ethylene[6] used in both the biogas- and DAC CO_2-based routes for propanol production, the values were directly taken from literature sources to ensure methodological consistency. The summation of CAPEX and OPEX values represent total annualized production costs and are expressed in USD per kg of propanol. All costs were reported in 2023 USD. When necessary, historical values were adjusted using the average chemical engineering plant cost index (CEPCI).

Table S7. Cost parameters used to calculate the unitary production cost of propanol with all costs reported using 2023 as the reference year.

Flow	Average	Low	High	Source
DAC CO_2 / USD kg^{-1}	0.362	0.269	0.623	[9]
Electrolytic hydrogen (wind-based) / USD kg^{-1}	6.072	5.075	7.128	[10]
Biogas / USD kg^{-1}	0.286	0.200	0.355	[11]
Natural gas / USD kg^{-1}	0.447	0.096	0.902	[12]
Fossil ethylene / USD kg^{-1}	0.908	0.585	1.164	[8]
Green (MTO-based) ethylene / USD kg^{-1}	4.236	4.192	4.281	[6]
Heating (natural gas) / USD GJ^{-1}	9.499	–	–	[12]
Cooling water / USD GJ^{-1}	0.500	–	–	[6]
Grid electricity / USD kWh^{-1}	0.086	–	–	[6]
Fossil propanol / USD kg^{-1}	1.340	0.890	1.530	[7]

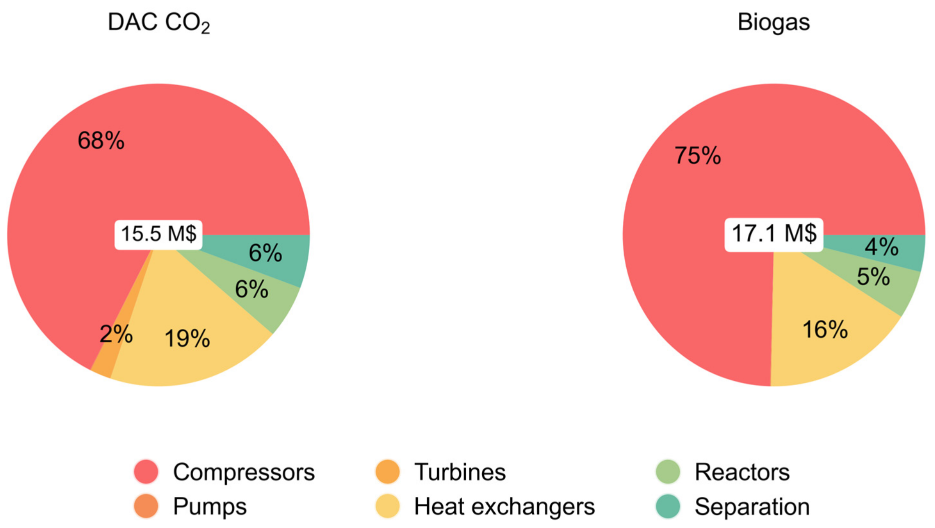

Figure S3. Breakdown of CAPEX for the DAC CO_2 and biogas-based propanol production routes. The total purchased equipment costs are displayed in the white rectangle at the center of each pie chart, expressed in million USD.

3. Life cycle assessment methodology

The global average climate change impacts of propanol production are quantified using an attributional life cycle assessment (LCA) conducted in accordance with the ISO 14040/14044 standards.[13,14] The production of 1 kg of n-propanol is defined as the functional unit, following a cradle-to-gate system boundary. This boundary encompasses all processes from feedstock acquisition to the production of 99.5 wt% pure propanol, while excluding the end-use phase. The end-use stage is omitted because it is assumed to be identical across all production routes and therefore adds no discriminatory power to the comparative analysis. Three production routes are considered: (i) the biogas route, (ii) the route utilizing DAC CO_2 and electrolytic hydrogen, and (iii) the fossil-based propanol route (reference case).

Detailed process flowsheets for the biogas and DAC CO_2 routes are shown in **Figures S1** and **S2**. The mass and energy balances for these two routes were obtained from Aspen Plus v12.1 simulations (**Table S1**) and serve as foreground data. The background system, representing upstream activities such as electricity generation, heat supply, and feedstock extraction, was modeled using datasets from the ecoinvent v3.10 database.[15] For the fossil-based propanol route, all inventory data were taken directly from ecoinvent v3.10. The complete life cycle inventories deployed for all production routes are summarized in **Table S3**.

The climate change impacts were quantified using the 100-year global warming potentials (GWPs) as reported by the IPCC.[16] In addition to climate change, other environmental impact categories, *i.e.*, human health, ecosystem quality, and resource depletion, were assessed using the ReCiPe 2016 v1.03 endpoint method.[17]

An uncertainty analysis was performed for all production routes using Monte Carlo sampling with 500 iterations. The ecoinvent pedigree matrix approach was used to parameterize uncertainties. Specifically, lognormal distributions were applied to background system data, following ecoinvent's default parameterization.

Additionally, we evaluated the contribution of propanol production to downstream products and activities, as reported in the main manuscript. These downstream activities and their respective impact contributions are summarized in **Table S4**. To ensure consistency, we replaced the "market for 1-propanol" activity in these downstream inventories with the fossil propanol production activity reported in **Table S3**. Subsequently, we quantified the change in propanol's impact contribution to these downstream activities, reflecting the low-carbon production routes analyzed in this study.

All calculations for the environmental assessment were performed using the Brightway2.5 framework.[18] To estimate the environmental impacts for the year 2050, a prospective LCA (pLCA) was conducted considering future energy system configurations. An optimistic future scenario aligned with the 2 °C climate target was assumed. The background system was updated using projections from integrated assessment models (IAMs), specifically the IMAGE model, which simulates interactions among the biosphere, society, and climate.[19] Future background data were constructed using the *premise* v2.1.3 framework.[20] We assume the shared socioeconomic pathway SSP2 ('middle-of-the-road') and the mitigation representative concentration pathway RCP2.6, corresponding to a global mean surface temperature increase limited to 2 °C. Notably, no uncertainty assessment was performed for the prospective scenarios due to the lack of available uncertainty data, *i.e.*, the corresponding pedigree matrix values.

Table S8. Inventories used in the LCA to calculate the impacts of all propanol production technologies.

Flow	Activity	Source
Fossil propanol	1-propanol production	ecoinvent v3.10
DAC CO_2	carbon dioxide capture, from atmosphere	Terlouw *et al.*[21]
Electrolytic hydrogen (wind-based)	hydrogen production, PEM electrolysis	D'Angelo *et al.*[22]
Biogas	market for biogas, sustainable feedstocks	Istrate *et al.*[1]
Fossil ethylene	market for ethylene	ecoinvent v3.10
Green (MTO) ethylene	ethylene production, green methanol-to-olefins	Ioannou *et al.*[6]
Monoethanolamine	market for monoethanolamine	ecoinvent v3.10
Tap water	market for tap water	ecoinvent v3.10
Wastewater treatment	market for wastewater, average	ecoinvent v3.10
Grid electricity	market group for electricity, high voltage	ecoinvent v3.10
Wind electricity	electricity production, wind, >3MW turbine, onshore	ecoinvent v3.10
Cooling water	cooling water	Ioannou *et al.*[6]
Heating (natural gas)	heat production, natural gas, at boiler condensing modulating >100kW	ecoinvent v3.10

Table S9. Downstream activities using propanol in ecoinvent v3.10. To calculate the climate change impact contribution of propanol to these activities, the 'market for 1-propanol' activity was replaced with the corresponding '1-propanol' production' activity, as reported in **Table S3**.

**Activity**	**% contribution of propanol**
propyl acetate production (kilogram, RER, None)	46.0
prochloraz production (kilogram, GLO, None)	13.5
propyl amine production (kilogram, RoW, None)	72.5
propyl acetate production (kilogram, RoW, None)	44.8
propyl amine production (kilogram, RER, None)	77.3

4. Assumptions and limitations of the study

- The pLCA study results are influenced by the assumptions embedded in the IMAGE IAM, which provides the background inventories.[19] To generate prospective background inventories, this study employs the *premise* python package.[20]
- Inventory data were obtained from ecoinvent v3.10 using the cut-off system model.[15] Global inventories were prioritized; when unavailable, data representing the rest of the world were applied.
- Life cycle assessments were performed for the production of 1 kg of propanol using multiple impact assessment methods.[16,17] All analyses followed a cradle-to-gate approach to avoid assumptions about the use phase of propanol and thus minimize associated uncertainties. The end-use phase was excluded, as it was assumed to be consistent across all scenarios and therefore offered no discriminatory power for comparative analysis.[23]
- The operation of compressors and pumps in the propanol production process was assumed to be continuous and powered by grid electricity. In contrast, the water electrolysis and the DAC units were assumed to run solely on onshore wind electricity, operating intermittently. A buffer storage tank ensures a steady supply to the production processes operating under continuous conditions.
- For electrolytic hydrogen production, only proton exchange membrane (PEM) water electrolysis powered by onshore wind electricity was considered, reflecting its strong potential for large-scale deployment.
- Data trends suggest that while end-of-life recycling could modestly reduce the carbon intensity of onshore wind turbines, the projected impact is minimal.[24] Consequently, no future efficiency improvements were assumed for these systems.
- The study primarily focuses on mature technologies, for which efficiency gains are assumed negligible. For instance, potential improvements in electrolyzer efficiency are excluded, since their contribution is minor compared to the overall decarbonization of the electricity grid.[25]
- Predictions derived from IAMs carry inherent limitations. The *premise* tool currently focuses on power generation, cement and steel production, transport, and fuels. Furthermore, uncertainties are not explicitly addressed in this analysis due to insufficient data in the future-modified background pedigree matrix of the ecoinvent database.
- At the production stage (cradle-to-gate scope), propanol, particularly in the DAC CO_2 and biogas scenarios with green ethylene, exhibits negative climate impacts due to CO_2 removal from the atmosphere or the incorporation of biogenic carbon. However, these negative values should not be interpreted as a carbon sink, since the CO_2 is not permanently stored within propanol over the long term.

5. Breakdown of climate change impacts in 2050

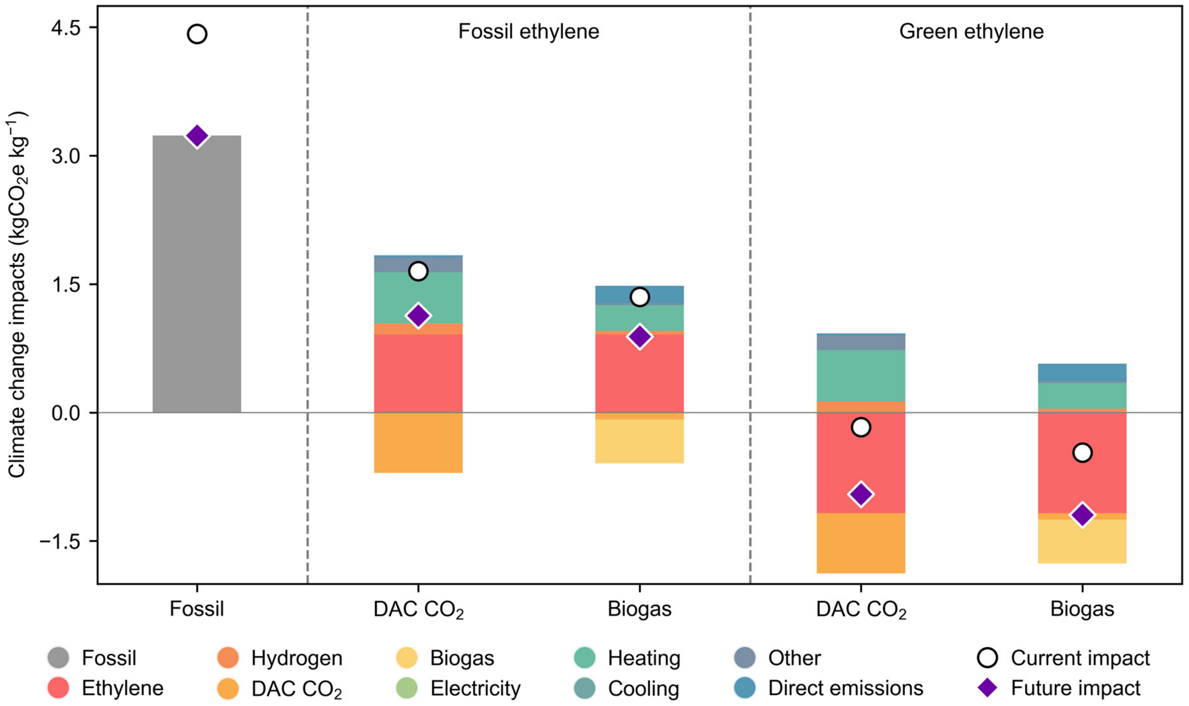

Figure S4. Climate change impacts by 2050 per kg of propanol produced using all considered technologies. Error bars for the climate change impacts are omitted due to insufficient data points in the *premise* database (**Section 4** of the SI).

6. Additional impact categories

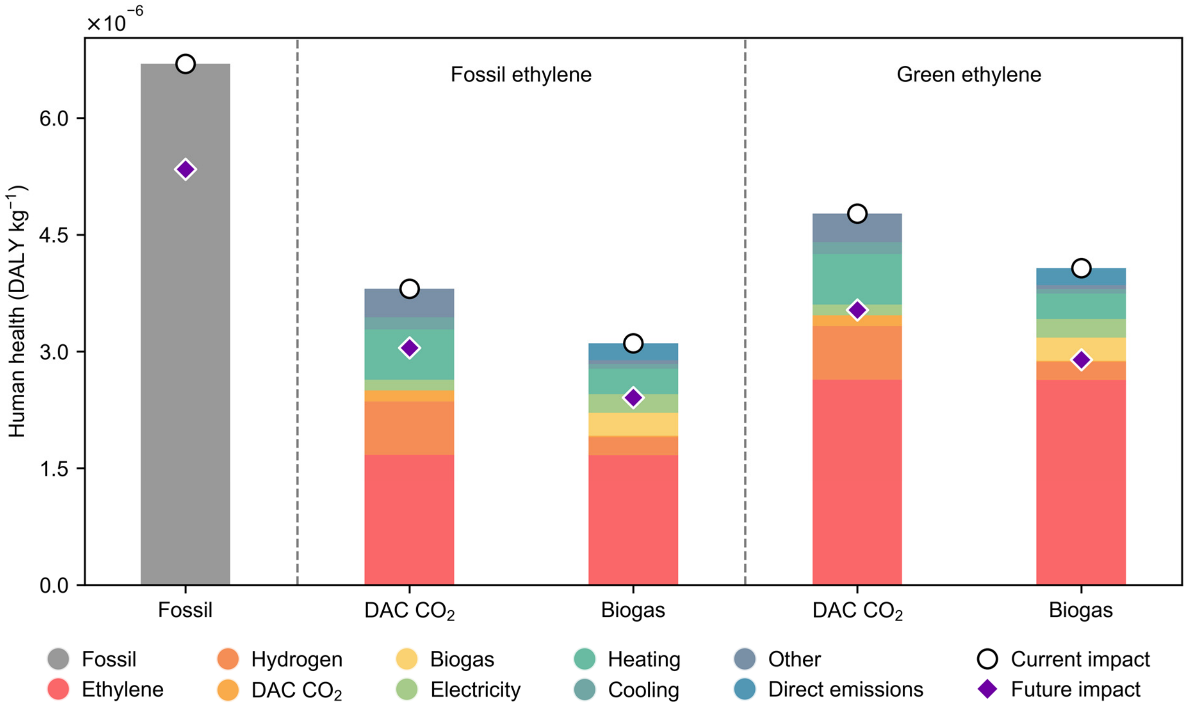

Figure S5. Breakdown of human health impacts for the assessed propanol production scenarios. The uncertainty calculated using Monte Carlo simulations is shown in **Figures S8–S10**.

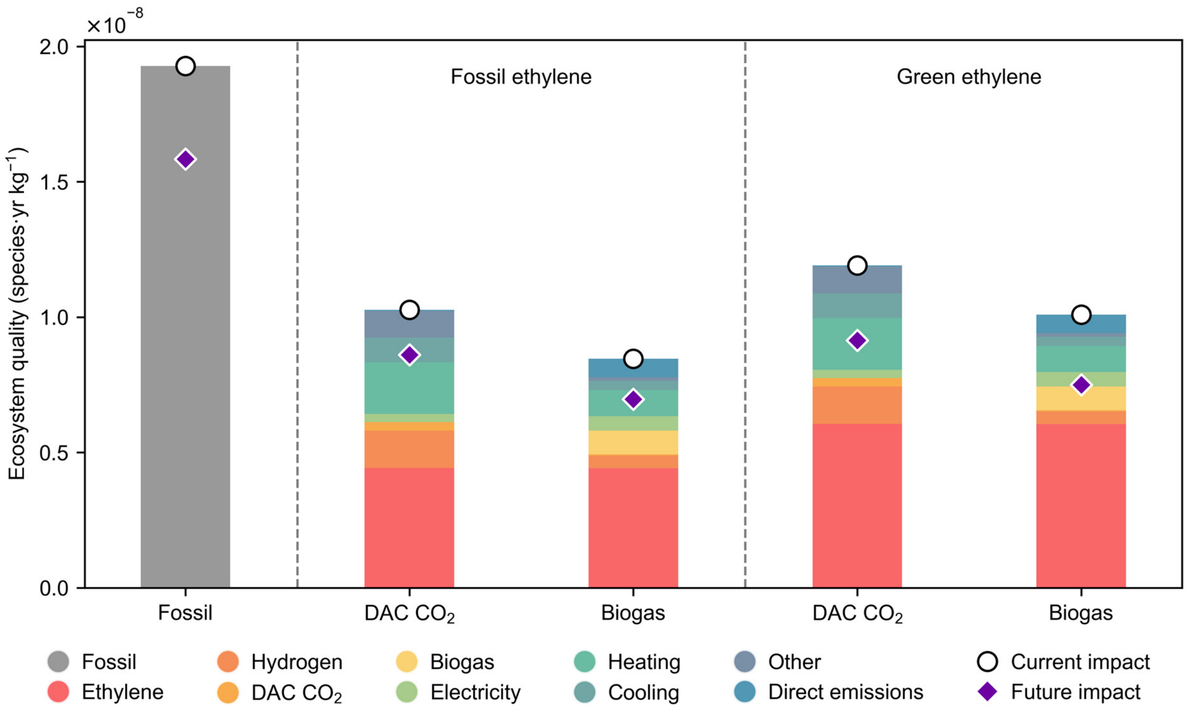

Figure S6. Breakdown of ecosystem quality impacts for the assessed propanol production scenarios. The uncertainty calculated using Monte Carlo simulations is shown in **Figures S8–S10**.

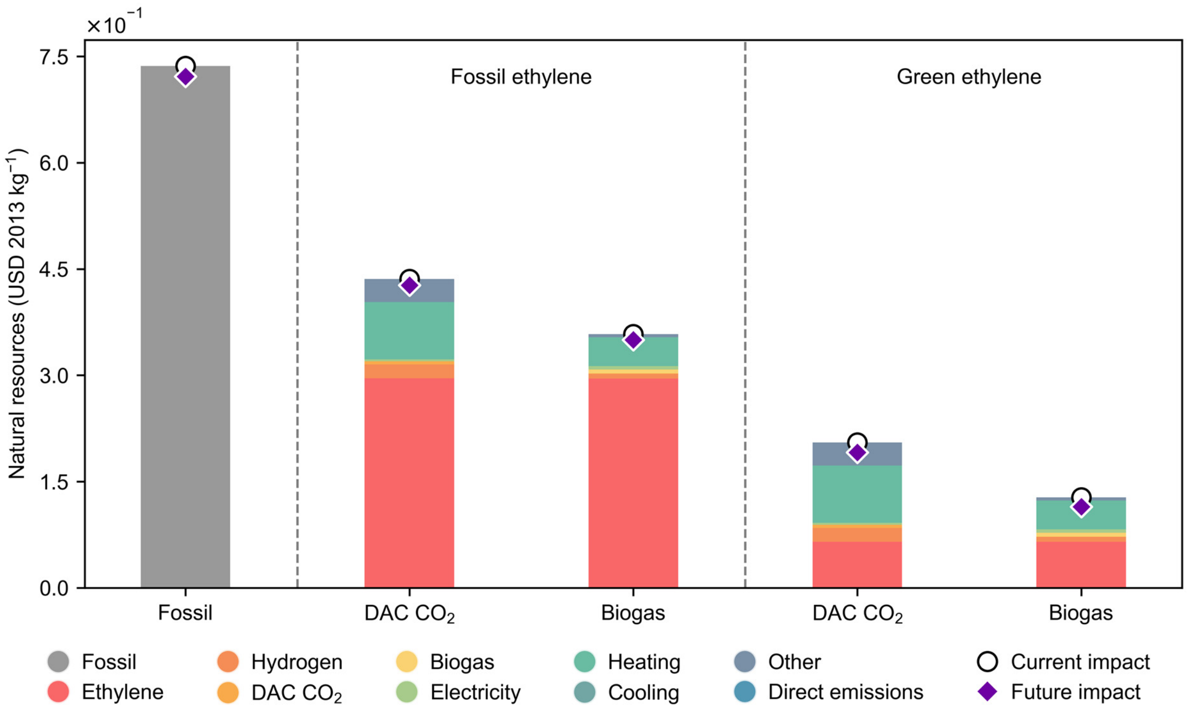

Figure S7. Breakdown of natural resources impact for the assessed propanol production scenarios. The uncertainty calculated using Monte Carlo simulations is shown in **Figures S8–S10**.

7. Uncertainty analysis

The uncertainty assessment was performed using Monte Carlo sampling based on the ecoinvent pedigree matrix values, which provide uncertainty data for the LCI values of the background system. This matrix incorporates various qualitative criteria, including geographical and temporal aspects, to model the uncertainty distribution of the LCI parameters in the ecoinvent database. The outcomes of the uncertainty assessment and the probability of burden shifting are presented in **Figures S8–S10**.

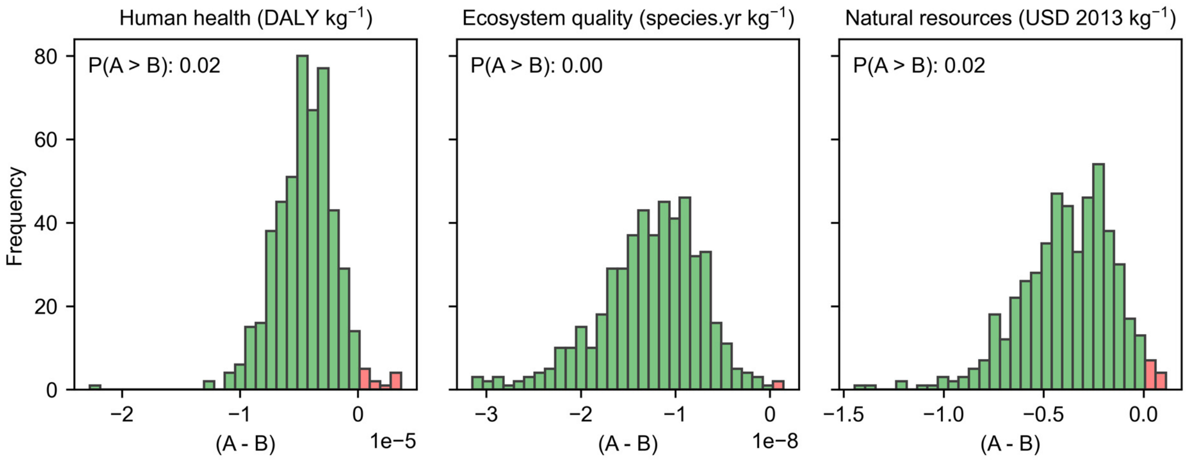

Figure S8. Probability of burden-shifting (A > B) for propanol production technologies across the three endpoint categories of the ReCiPe 2016 v1.03 method. Here, A represents the DAC CO_2-based propanol production route utilizing fossil ethylene, and B represents the fossil-based business-as-usual propanol production route. Green bars indicate cases in which the environmental footprint of the fossil process is lower than that of the biogas-based process, whereas red bars indicate cases in which the environmental footprint of the fossil scenario is higher. Burden shifting is considered to occur when P(A > B) ≥ 0.75, while no burden shifting is observed when P(A > B) < 0.25.

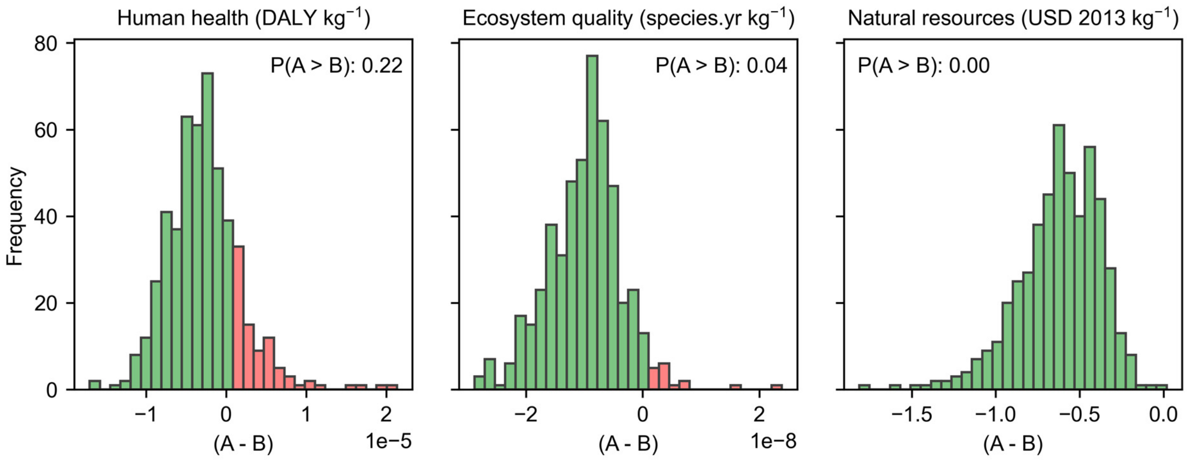

Figure S9. Probability of burden-shifting (A > B) for propanol production technologies across the three endpoint categories of the ReCiPe 2016 v1.03 method. Here, A represents the DAC CO_2-based propanol production route utilizing green ethylene, and B represents the fossil-based business-as-usual propanol production route. Green bars indicate cases in which the environmental footprint of the fossil process is lower than that of the biogas-based process, whereas red bars indicate cases in which the environmental footprint of the fossil scenario is higher. Burden shifting is considered to occur when P(A > B) ≥ 0.75, while no burden shifting is observed when P(A > B) < 0.25.

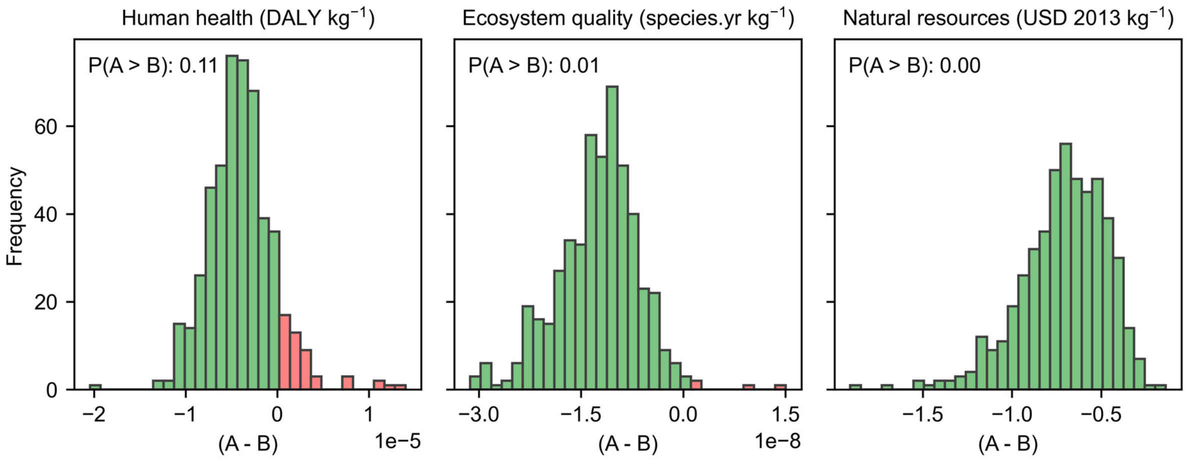

Figure S10. Probability of burden-shifting (A > B) for propanol production technologies across the three endpoint categories of the ReCiPe 2016 v1.03 method. Here, A represents the biogas-based propanol production route utilizing green ethylene, and B represents the fossil-based business-as-usual propanol production route. Green bars indicate cases in which the environmental footprint of the fossil process is lower than that of the biogas-based process, whereas red bars indicate cases in which the environmental footprint of the fossil scenario is higher. Burden shifting is considered to occur when P(A > B) ≥ 0.75, while no burden shifting is observed when P(A > B) < 0.25.

References

(1) Istrate, R.; Nabera, A.; Pérez-Ramírez, J.; Guillén-Gosálbez, G. One-Tenth of the EU's Sustainable Biomethane Coupled with Carbon Capture and Storage Can Enable Net-Zero Ammonia Production. *One Earth* **2024**, *7* (12), 2235–2249. https://doi.org/10.1016/j.oneear.2024.11.005.

(2) Medrano-García, J. D.; Ruiz-Femenia, R.; Caballero, J. A. Multi-Objective Optimization of Combined Synthesis Gas Reforming Technologies. *J. CO_2 Util.* **2017**, *22*, 355–373. https://doi.org/10.1016/j.jcou.2017.09.019.

(3) Vo, C. H.; Mondelli, C.; Hamedi, H.; Pérez-Ramírez, J.; Farooq, S.; Karimi, I. A. Sustainability Assessment of Thermocatalytic Conversion of CO_2 to Transportation Fuels, Methanol, and 1-Propanol. *ACS Sustain. Chem. Eng.* **2021**, *9* (31), 10591–10600. https://doi.org/10.1021/acssuschemeng.1c02805.

(4) Medrano-García, J. D.; Chagas, M. T.; Guillén-Gosálbez, G. Integrating the Reverse Boudouard Reaction for a More Efficient Green Methanol Synthesis from CO_2 and Renewable Energy. *ACS Sustain. Chem. Eng.* **2025**, *13* (19), 7088–7097. https://doi.org/10.1021/acssuschemeng.5c01021.

(5) Towler, G. P.; Sinnott, R. K. *Sinnott & Towler*; Butterworth-Heinemann, 2013.

(6) Ioannou, I.; D'Angelo, S. C.; Martín, A. J.; Pérez-Ramírez, J.; Guillén-Gosálbez, G. Hybridization of Fossil- and CO_2-Based Routes for Ethylene Production Using Renewable Energy. *ChemSusChem* **2020**, *13* (23), 6370–6380. https://doi.org/10.1002/cssc.202001312.

(7) Business Analytiq. *nPropanol (1-propanol) price index*. https://businessanalytiq.com/procurementanalytics/index/npropanol-price-index/ (accessed 2025-10-18).

(8) Business Analytiq. *Ethylene price index*. https://businessanalytiq.com/procurementanalytics/index/ethylene-price-index/ (accessed 2025-10-18).

(9) Young, J.; McQueen, N.; Charalambous, C.; Foteinis, S.; Hawrot, O.; Ojeda, M.; Pilorgé, H.; Andresen, J.; Psarras, P.; Renforth, P.; Garcia, S.; Van Der Spek, M. The Cost of Direct Air Capture and Storage Can Be Reduced via Strategic Deployment but Is Unlikely to Fall below Stated Cost Targets. *One Earth* **2023**, *6* (7), 899–917. https://doi.org/10.1016/j.oneear.2023.06.004.

(10) Nabera, A.; Istrate, I.-R.; Martín, A. J.; Pérez-Ramírez, J.; Guillén-Gosálbez, G. Energy Crisis in Europe Enhances the Sustainability of Green Chemicals. *Green Chem.* **2023**, *25* (17), 6603–6611. https://doi.org/10.1039/D3GC01053H.

(11) IEA. *Outlook for Biogas and Biomethane: Prospects for Organic Growth*; OECD, 2020. https://doi.org/10.1787/040c8cd2-en.

(12) World Bank. *Monthly Prices, Commodity Markets*. World Bank.

(13) International Standards Organization. *In ISO 14040:2006 Environmental Management--Life Cycle Assessment--Principles and Framework*; 2006.

(14) International Standards Organization. *In ISO 14044:2006 Environmental Management--Life Cycle Assessment--Requirements and Guidelines*; 2006.

(15) Wernet, G.; Bauer, C.; Steubing, B.; Reinhard, J.; Moreno-Ruiz, E.; Weidema, B. The Ecoinvent Database Version 3 (Part I): Overview and Methodology. *Int. J. Life Cycle Assess.* **2016**, *21* (9), 1218–1230. https://doi.org/10.1007/s11367-016-1087-8.
(16) Accelerating the Transition in the Context of Sustainable Development. In *Climate Change 2022 - Mitigation of Climate Change*; Intergovernmental Panel on Climate Change (IPCC), Ed.; Cambridge University Press, 2023; pp 1727–1790. https://doi.org/10.1017/9781009157926.019.
(17) Huijbregts, M. A. J.; Steinmann, Z. J. N.; Elshout, P. M. F.; Stam, G.; Verones, F.; Vieira, M.; Zijp, M.; Hollander, A.; van Zelm, R. ReCiPe2016: A Harmonised Life Cycle Impact Assessment Method at Midpoint and Endpoint Level. *Int. J. Life Cycle Assess.* **2017**, *22* (2), 138–147. https://doi.org/10.1007/s11367-016-1246-y.
(18) Mutel, C. Brightway: An Open Source Framework for Life Cycle Assessment. *J. Open Source Softw.* **2017**, *2* (12), 236. https://doi.org/10.21105/joss.00236.
(19) *Integrated Assessment of Global Environmental Change with IMAGE 3.0*; PBL Netherlands Environmental Assessment Agency, 2014.
(20) Sacchi, R.; Terlouw, T.; Siala, K.; Dirnaichner, A.; Bauer, C.; Cox, B.; Mutel, C.; Daioglou, V.; Luderer, G. PRospective EnvironMental Impact asSEment (Premise): A Streamlined Approach to Producing Databases for Prospective Life Cycle Assessment Using Integrated Assessment Models. *Renew. Sustain. Energy Rev.* **2022**, *160*, 112311. https://doi.org/10.1016/j.rser.2022.112311.
(21) Terlouw, T.; Treyer, K.; Bauer, C.; Mazzotti, M. Life Cycle Assessment of Direct Air Carbon Capture and Storage with Low-Carbon Energy Sources. *Environ. Sci. Technol.* **2021**, *55* (16), 11397–11411. https://doi.org/10.1021/acs.est.1c03263.
(22) D'Angelo, S. C.; Cobo, S.; Tulus, V.; Nabera, A.; Martín, A. J.; Pérez-Ramírez, J.; Guillén-Gosálbez, G. Planetary Boundaries Analysis of Low-Carbon Ammonia Production Routes. *ACS Sustain. Chem. Eng.* **2021**, *9* (29), 9740–9749. https://doi.org/10.1021/acssuschemeng.1c01915.
(23) Galán-Martín, Á.; Tulus, V.; Díaz, I.; Pozo, C.; Pérez-Ramírez, J.; Guillén-Gosálbez, G. Sustainability Footprints of a Renewable Carbon Transition for the Petrochemical Sector within Planetary Boundaries. *One Earth* **2021**, *4* (4), 565–583. https://doi.org/10.1016/j.oneear.2021.04.001.
(24) Besseau, R.; Sacchi, R.; Blanc, I.; Pérez-López, P. Past, Present and Future Environmental Footprint of the Danish Wind Turbine Fleet with LCA_WIND_DK, an Online Interactive Platform. *Renew. Sustain. Energy Rev.* **2019**, *108*, 274–288. https://doi.org/10.1016/j.rser.2019.03.030.
(25) Nabera, A.; José Martín, A.; Istrate, R.; Pérez-Ramírez, J.; Guillén-Gosálbez, G. Integrating Climate Policies in the Sustainability Analysis of Green Chemicals. *Green Chem.* **2024**, *26* (11), 6461–6469. https://doi.org/10.1039/D4GC00392F.
